# Brain network analysis reveals that amyloidopathy affects comorbid cognitive dysfunction in older adults with depression

**DOI:** 10.1038/s41598-021-83739-3

**Published:** 2021-02-22

**Authors:** Suji Lee, Daegyeom Kim, HyunChul Youn, Won Seok William Hyung, Sangil Suh, Marcus Kaiser, Cheol E. Han, Hyun-Ghang Jeong

**Affiliations:** 1 Department of Biomedical Sciences, Korea University Graduate School, Seoul, Republic of Korea; 2 Department of Electronics and Information Engineering, Korea University, Sejong, Republic of Korea; 3 Department of Psychiatry, Soonchunhyang University Bucheon Hospital, Bucheon, Republic of Korea; 4 Department of Psychiatry, Korea University Guro Hospital, Korea University College of Medicine, Seoul, Republic of Korea; 5 Department of Radiology, Korea University Guro Hospital, Korea University College of Medicine, Seoul, Republic of Korea; 6 Interdisciplinary Computing and Complex BioSystems (ICOS) Research Group, School of Computing, Newcastle University, Newcastle upon Tyne, NE4 5TG UK; 7 Institute of Neuroscience, Newcastle University, The Henry Wellcome Building, Newcastle upon Tyne, NE2 4HH UK; 8 Department of Functional Neurosurgery, School of Medicine, Ruijin Hospital, Shanghai Jiao Tong University, Shanghai, 200025 China; 9 Precision Imaging Beacon, School of Medicine, University of Nottingham, Nottingham, NG7 2UH UK; 10 Interdisciplinary Graduate Program for Artificial Intelligence Smart Convergence Technology, Korea University, Sejong, Republic of Korea

**Keywords:** Neuroscience, Medical research

## Abstract

Late-life depression (LLD) may increase the risk of Alzheimer’s dementia (AD). While amyloidopathy accelerates AD progression, its role in such patients has not yet been elucidated. We hypothesized that cerebral amyloidopathy distinctly affects the alteration of brain network topology and may be associated with distinct cognitive symptoms. We recruited 26 and 27 depressed mild cognitive impairment (MCI) patients with (LLD-MCI-A(+)) and without amyloid accumulation (LLD-MCI-A(−)), respectively, and 21 normal controls. We extracted structural brain networks using their diffusion-weighted images. We aimed to compare the distinct network deterioration in LLD-MCI with and without amyloid accumulation and the relationship with their distinct cognitive decline. Thus, we performed a group comparison of the network topological measures and investigated any correlations with neurocognitive testing scores. Topological features of brain networks were different according to the presence of amyloid accumulation. Disrupted network connectivity was highly associated with impaired recall and recognition in LLD-MCI-A(+) patients. Inattention and dysexecutive function were more influenced by the altered networks involved in fronto-limbic circuitry dysfunction in LLD-MCI-A(−) patients. Our results show that alterations in brain network topology may reflect different cognitive dysfunction depending on amyloid accumulation in depressed older adults with MCI.

## Introduction

Late-life depression (LLD) is often found together with mild cognitive impairment (MCI), which is often considered the prodromal stage of Alzheimer’s disease (AD). Epidemiological studies have revealed that older adults with prodromal AD have a higher frequency of depression^1,2^. At the same time, depressed older adults without AD pathology also frequently have concomitant cognitive impairment^3^. Therefore, it is a common clinical phenotype for older adults to present with both LLD and MCI. However, they exhibit different prognoses depending on the heterogeneity of the underlying pathogenesis. For example, some of them progress to dementia, some stay in the MCI stage, and some recover to their normal condition^4^. Thus, it is critical to detect the underlying neuropathology among such individuals before further cognitive deterioration progression.

Our earlier studies demonstrated that cerebral amyloidopathy, one of the core AD pathologies, may contribute to impairments in distinct cognitive domains in patients with LLD and MCI^3,5,6^. Specifically, subjects with amyloidopathy had lower word-list recall and constructional recall scores than those without amyloidopathy^3^. In another report, we further noticed different regional glucose metabolism between those with and without cerebral amyloidopathy^5^. In particular, individuals with amyloid accumulation had a decreased level of regional cerebral glucose metabolism in the temporoparietal region, which is highly associated with memory function. In contrast, individuals without amyloid accumulation had cerebral glucose hypometabolism within the frontal region, which is responsible for executive function. These results suggest that cerebral amyloid accumulation possibly determines distinguished cognitive impairment and altered brain metabolism in patients with LLD and MCI.

Given that patients with LLD and MCI present a broad spectrum of clinical symptoms related to cognitive dysfunction and emotional disturbances based on the pathophysiological mechanisms, it is postulated that they may be mediated by widespread network disconnectivity rather than by a single brain region^7,8^. In previous research, a network analysis based on combining magneto-encephalography showed that dysfunctional communication between the brain modules resulted in cognitive impairment in patients with AD, indicating the significance of investigating the global network organization^9^. In another recent study that investigated cognitively intact individuals with abnormal amyloid levels of cerebrospinal fluid (CSF), it was observed from cortical thickness data that cerebral amyloidopathy influenced the disruption of global connectivity across the brain network^10^. Its authors also showed that healthy subjects with abnormal CSF Aβ42/Aβ40 levels and no apparent cerebral atrophy had a lower degree of global efficiency than those without Aβ accumulation. These findings suggest that the various symptoms are mediated by a widespread network of disconnectivity under the influence of neuropathology, even in the preclinical or prodromal phase of AD. However, it is still unclear how different brain network regions interact with one another to produce cognitive and depressive symptoms and whether specific alterations in brain network topology reflect the presence or absence of underlying neuropathological changes.

Therefore, we utilized a network analysis derived from diffusion-weighted imaging (DWI) to investigate whether amyloid accumulation influences brain network topology in older adults with concomitant depression and MCI. In addition, the relationship between network properties and performance on neurocognitive tests were also analyzed. We hypothesized that alteration in the brain network in the temporoparietal area and AD-related regions would be linked to cognitive impairment in patients with amyloid accumulation. On the other hand, in subjects without amyloid accumulation, an altered network in the fronto-limbic area would be found to be associated with their cognitive symptoms.

## Results

### Subject characteristics

Table 1 shows the characteristics and cognitive scores of all of the participants. We performed an analysis of variance (ANOVA) to investigate the difference between the groups' cognitive scores. Group differences existed in all applied cognitive examinations except for the constructional praxis z-score and trail-making test A (Table 1). We also performed post-hoc tests for each score, which showed that the variations between healthy older adults (HOA) and depressed MCI patients without amyloid accumulation (LLD-MCI-A(−)) and those between HOA and depressed MCI patients with amyloid accumulation (LLD-MCI-A(+)) were significant.

**Table 1:** Demographic information.

Item	HOA	LLD-MCI-A(+)	LLD-MCI-A(−)	Three-group comparison	HOA vs. LLD-MCI-A(+)	HOA vs. LLD-MCI-A(−)	LLD-MCI-A(+) vs. LLD-MCI-A(−)
Age	68.86 ± 4.60^a^	76.85 ± 7.30^a^	76.07 ± 5.41^a^	F = 12.356, P < 0.001	t = − 4.359, P < 0.001	t = − 4.892, P < 0.001	t = 0.439; P = 0.663
Sex (male/female)	12/9	4/22	2/25	χ^2^ = 17.614^b^, P < 0.001	χ^2^ = 9.022^b^, P = 0.005	χ^2^ = 14.143^b^, P < 0.001	χ^2^ = 0.839^b^, P = 0.360
Education	13.33 ± 4.23	6.92 ± 4.42	6.37 ± 4.36	F = 17.940, P < 0.001	t = 5.037, P < 0.001	t = 5.557, P < 0.001	t = 0.458; P = 0.649
Geriatric Depression Scale	8.71 ± 4.22	13.12 ± 6.78	15.70 ± 6.37	F = 8.060, P < 0.001	t = − 2.594, P = 0.013	t = − 4.339, P < 0.001	t = − 1.434, P = 0.158
MMSE	28.95 ± 0.92	20.88 ± 3.86	22.74 ± 4.85	F = 28.682, P < 0.001	t = 9.345, P < 0.001	t = 5.777, P < 0.001	t = − 1.538; P = 0.130
Word-list learning	21.86 ± 3.18	9.96 ± 3.40	11.63 ± 4.10	F = 71.624, P < 0.001	t = 12.268, P < 0.001	t = 9.432, P < 0.001	t = − 1.610; P = 0.114
Word-list learning z-score	1.31 ± 0.76	− 1.36 ± 0.65	− 0.98 ± 0.83	F = 83.036, P < 0.001	t = 12.958, P < 0.001	t = 9.805, P < 0.001	t = − 1.843; P = 0.071
Word-list recall	7.76 ± 1.45	1.38 ± 1.42	2.63 ± 2.06	F = 90.673, P < 0.001	t = 15.205, P < 0.001	t = 9.700, P < 0.001	t = 2.555; P = 0.014
Word-list recall z-score	0.93 ± 0.74	2.05 ± 0.64	− 1.42 ± 0.88	F = 95.745, P < 0.001	t = 14.783, P < 0.001	t = 9.819, P < 0.001	t = 2.958; P = 0.005
Word-list recognition	9.57 ± 0.81	5.58 ± 3.00	6.22 ± 2.99	F = 15.677, P < 0.001	t = 5.926, P < 0.001	t = 4.980, P < 0.001	t = − 0.785; P = 0.436
Word-list recognition z-score	0.39 ± 0.59	− 1.88 ± 1.97	− 1.43 ± 1.72	F = 12.829, P < 0.001	t = 5.088, P < 0.001	t = 4.632, P < 0.001	t = − 0.896; P = 0.375
Constructional praxis	10.14 ± 0.96	8.58 ± 2.37	7.96 ± 1.83	F = 8.385, P < 0.001	t = 2.840; P = 0.007	t = 4.946, P < 0.001	t = 1.058; P = 0.295
Constructional praxis z-score	− 0.06 ± 0.72	0.04 ± 0.88	− 0.53 ± 1.48	F = 1.976; P = 0.146	t = − 0.454; P = 0.652	t = 1.315; P = 0.292	t = 1.698; P = 0.287
Constructional praxis recall	7.43 ± 2.42	1.58 ± 1.98	2.81 ± 2.20	F = 44.722, P < 0.001	t = 9.114, P < 0.001	t = 6.896, P < 0.001	t = 2.148; P = 0.037
Constructional praxis recall z-score	0.086 ± 0.84	− 1.48 ± 0.93	− 0.64 ± 0.95	F = 17.236, P < 0.001	t = 5.970, P < 0.001	t = 2.774; P = 0.008	t = − 3.236; P = 0.003
Trail-making test A	1.00 ± 0	0.85 ± 0.37	0.78 ± 0.42	F = 2.634; P = 0.079	t = 1.912; P = 0.093	t = 2.398; P = 0.062	t = − 0.626; P = 0.534
Trail-making test A z-score	1.38 ± 0.63	− 0.01 ± 0.99	− 0.66 ± 1.46	F = 19.426, P < 0.001	t = 5.458, P < 0.001	t = 5.858, P < 0.001	t = 1.695; P = 0.098
Trail-making test A (seconds)	40.48 ± 12.90	113.14 ± 58.22	138.14 ± 86.32	F = 14.784, P < 0.001	t = − 5.587, P < 0.001	t = − 5.128, P < 0.001	t = − 1.118; P = 0.270
Boston naming	12.76 ± 1.58	9.23 ± 3.01	8.70 ± 2.84	F = 16.128, P < 0.001	t = 4.856, P < 0.001	t = 5.873, P < 0.001	t = 0.656; P = 0.515
Boston naming z-score	0.75 ± 0.61	− 0.08 ± 0.95	− 0.17 ± 0.84	F = 8.649, P < 0.001	t = 3.469; P = 0.001	t = 4.265, P < 0.001	t = 0.395; P = 0.694
Digit-span forward test	6.52 ± 1.25	4.46 ± 0.90	4.00 ± 0.83	F = 41.870, P < 0.001	t = 6.558, P < 0.001	t = 8.384, P < 0.001	t = 1.934; P = 0.059
Digit-span backward test	4.47 ± 1.17	2.73 ± 0.67	2.63 ± 0.88	F = 29.478, P < 0.001	t = 6.444, P < 0.001	t = 6.243, P < 0.001	t = 0.469; P = 0.641
COWAT^c^ animal	16.71 ± 4.28	8.77 ± 2.75	9.56 ± 3.33	F = 36.372, P < 0.001	t = 7.712, P < 0.001	t = 6.521, P < 0.001	t = − 0.935; P = 0.354
COWAT^c^ animal z-score	0.06 ± 0.86	− 1.27 ± 0.67	− 1.09 ± 0.89	F = 17.875, P < 0.001	t = 5.952, P < 0.001	t = 4.503, P < 0.001	t = − 0.835; P = 0.408
COWAT^c^ market	20.57 ± 6.66	10.88 ± 4.26	10.59 ± 4.26	F = 28.523, P < 0.001	t = 6.051, P < 0.001	t = 6.308, P < 0.001	t = 0.250; P = 0.804
COWAT^c^ market z-score	0.38 ± 1.20	− 0.65 ± 0.77	− 0.67 ± 0.93	F = 8.872, P < 0.001	t = 3.583; P = 0.001	t = 3.446; P = 0.001	t = 0.089; P = 0.929
COWAT^c^ ㄱ	9.38 ± 3.61	4.65 ± 3.23	2.67 ± 2.82	F = 25.332, P < 0.001	t = 4.586, P < 0.001	t = 6.991, P < 0.001	t = 2.247; P = 0.030
COWAT^c^ ㅇ	10.24 ± 4.09	4.35 ± 3.20	2.79 ± 2.87	F = 29.380, P < 0.001	t = 5.349, P < 0.001	t = 7.140, P < 0.001	t = 1.756; P = 0.086
COWAT^c^ ㅅ	10.19 ± 4.34	5.17 ± 3.70	3.17 ± 3.09	F = 20.889, P < 0.001	t = 4.135, P < 0.001	t = 6.311, P < 0.001	t = 2.022; P = 0.049

^a^Mean ± standard deviation.

^b^Yates’χ^2^.

^c^*COWAT* controlled oral word association test.

However, only parts of the scores between the MCI groups were significantly different, including the word-list recall score and its z-score, the constructional praxis recall score, and its z-score, controlled oral word association test (COWAT) lexicalㄱ, and COWAT lexicalㅅ. Furthermore, the average age, gender, and education level were not different between the LLD-MCI-A(−) and LLD-MCI-A(+) groups, while they were different from those of the HOA group (see limitations). There was no significant difference of depressive symptoms between LLD-MCI-A(+) and LLD-MCI-A(−) (t = − 1.434, P = 0.158).

### Group difference in network measures

**Figure 1:**
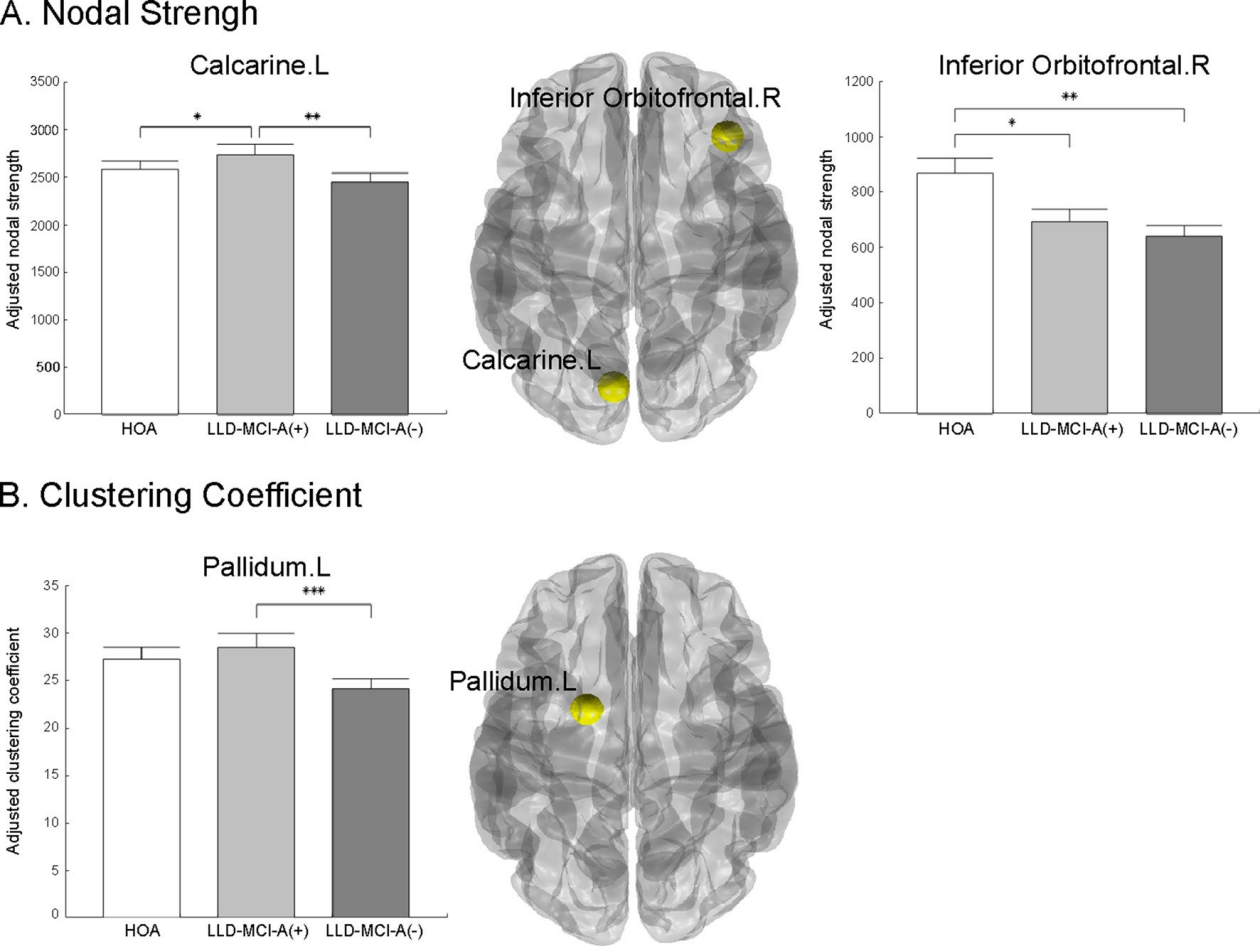
Between-group differences among the network measures. A between-group difference was found in the nodal strength (**A**) and clustering coefficient (**B**). Yellow circles in the middle brain images indicate the brain regions that show a group difference in the network measures. Bar graphs show the adjusted average values of the network measures in each group, controlling for age, gender, and education level. Whiskers of the bar graphs show the confidence intervals of the adjusted average values. All P-values were adjusted through the FDR procedure. *P < 0.05; **P < 0.01; ***P < 0.001.

We first investigated the group difference in the global network measures and did not find any significant difference even with uncorrected P-values under the alpha level of 0.05 (Table S1). For the nodal measures (Table S2–S6), we used the permutation-based analysis of covariance (ANCOVA) for the three groups and conducted a false-discovery rate (FDR) across 90 brain regions. Among the four nodal measures (nodal degree, nodal strength, nodal clustering coefficient, and regional efficiency), there were survived results in nodal strengths and nodal clustering coefficients after the FDR procedure. The nodal strength was significantly different between the groups in the left calcarine (F = 9.1496, FDR-adjusted P = 0.009; unless noted, the following P-values are FDR-adjusted P-values) and the right inferior orbitofrontal cortex (F = 8.1036, P = 0.036). The post-hoc tests showed that the two brain regions presented different patterns. The nodal strength of the left calcarine showed differences between the HOA and LLD-MCI-A(+) groups (F = 5.5690, P = 0.0324, mean ± standard deviation, HOA: 2580 ± 196, LLD-MCI-A(+): 2731 ± 289) and between the LLD-MCI-A(+) and LLD-MCI-A(−) groups (F = 12.8219, P = 0.0021, LLD-MCI-A(+): 2731 ± 289, LLD-MCI-A(−): 2442 ± 258). In contrast, the nodal strength of the right inferior orbitofrontal cortex significantly decreased in the LLD-MCI-A(+) (F = 6.3591, P = 0.0244) and LLD-MCI-A(−) (F = 14.4729, P = 0.0012) groups compared to the HOA (HOA: 868 ± 126, LLD-MCI-A(+): 692 ± 120, LLD-MCI(−): 640 ± 102). The clustering coefficient of the left pallidum significantly decreased in the LLD-MCI-A(−) group compared to the LLD-MCI-A(+) group (F = 25.4681, P = 0.0003, LLD-MCI-A(+): 28 ± 4, LLD-MCI-A(−): 24 ± 3; Fig. 1).

### Correlation between network measures and cognitive domains

We investigated the correlation between the network measures and the cognitive domain scores, controlling for age, gender, and education level. A correlation analysis was performed for the five global measures (i.e., total strength, edge density, small-worldness, characteristic path length (CPL), and clustering coefficient) and the four nodal measures (i.e., nodal degree, nodal strength, clustering coefficient node, and regional efficiency). Here, we report only the results that remained after the FDR procedure was conducted among the 90 brain regions (Tables 2, 3). For more detailed results, see the supplementary materials.

**Table 2:** Correlation between the global network measures and cognitive scores (significant results only).

Cognitive domain	Score	Network measure	LLD-MCI-A(+)		LLD-MCI-A(−)	
			r-value^a^	P-value^a^	r-value^a^	P-value^a^
Attention	Digit span test forward	Clustering coefficient			0.318	0.033
	Trail-making test A z -score	Total strength			0.571	< 0.001
		Edge density			0.377	0.018
		Clustering coefficient			0.388	0.015
		Characteristic path length			− 0.467	0.003
		Small-worldness			0.437	0.005
	Trail-making test A (seconds)	Total strength			− 0.519	0.001
		Edge density			− 0.391	0.014
		Characteristic path length			0.510	0.001
		Small-worldness			− 0.359	0.025
Recall and recognition	Word-list recall	Total strength	0.395	0.008		
		Characteristic path length	− 0.363	0.015		
	Word-list recall z-score	Total strength	0.375	0.012		
		Characteristic path length	− 0.319	0.035		
	Word-list recognition	Total strength	0.366	0.015		
		Characteristic path length	− 0.429	0.006		
	Word-list recognition z-score	Total strength	0.370	0.013		
		Characteristic path length	− 0.407	0.006		
Visuospatial function	Constructional praxis	Total strength	− 0.343	0.023	0.314	0.036
		Clustering coefficient	− 0.369	0.014		
		Characteristic path length			− 0.305	0.042
		Small-worldness	− 0.353	0.019		
	Constructional praxis z-score	Total strength	− 0.465	0.001		
		Clustering coefficient	− 0.307	0.043		
		Characteristic path length	0.413	0.005		
		Small-worldness	− 0.378	0.011		

^a^Partial correlation coefficient, controlling for age, gender, and education level.

**Table 3:** Correlation between the nodal network measures and cognitive scores (FDR-survived results only).

Cognitive domain	Score	Network measure	Node	LLD-MCI-A(+)		LLD-MCI-A(−)	
				r-value^a^	P-value^a^	r-value^a^	P-value^a^
Attention	Trail-making test A (seconds)	Clustering coefficient (node)	Right caudate	0.514	0.049		
		Regional efficiency	Left middle cingulum			− 0.539	0.028
Executive function	COWAT market	Nodal strength	Left inferior orbitofrontal			0.510	0.031
	COWAT market z-score	Clustering coefficient (node)	Right thalamus			− 0.500	0.042
		Regional efficiency	Left inferior orbitofrontal			0.495	0.049
	COWAT O	Clustering coefficient (node)	Right middle temporal			0.569	0.007
Learning	Word-list learning	Clustering coefficient (node)	Left middle occipital			− 0.533	0.015
	Word-list learning z-score	Nodal strength	Right inferior orbitofrontal			0.517	0.025
		Clustering coefficient (node)	Left middle occipital			− 0.541	0.011
Recall and recognition	Word-list recall	Regional efficiency	Right middle cingulum	0.498	0.028		
	Word-list recall z-score	Regional efficiency	Right middle cingulum	0.477	0.046		
Visuospatial function	Construction praxis recall	Regional efficiency	Right superior orbitofrontal			0.495	0.049

^a^Partial correlation coefficient, controlling for age, gender, and education level; the collected P-values were FDR-adjusted across 90 nodes.

For the attention and executive function domain, most of the significant correlations were found in the LLD-MCI-A(−) group. The digit span forward test was positively correlated with the clustering coefficient (r = 0.318). The trail-making test A (z-score) was correlated with total strength (r = 0.571), edge density (r = 0.377), clustering coefficient (r = 0.388), CPL (r = − 0.467), and small-worldness (r = 0.437). The trail-making test A (seconds) was correlated with total strength (r = − 0.519), edge density (r = − 0.391), CPL (r = 0.510), and small-worldness (r = − 0.359). Correlations among the nodal measures were observed for the trail-making A (seconds), COWAT market, COWAT market z-score, and COWAT lexicalㅇ evaluations. The trail-making A (seconds) test was negatively correlated with the left middle cingulum’s regional efficiency in the LLD-MCI-A(−) group (r = − 0.539), whereas it was positively correlated with the right caudate’s clustering coefficient in the LLD-MCI-A(+) group (r = 0.514). The COWAT market was positively correlated with the left inferior orbitofrontal cortex’s nodal strength (r = 0.510). The COWAT market z-score was further correlated with the right thalamus’s clustering coefficient (r = − 0.500) and the left inferior orbitofrontal cortex’s regional efficiency (r = 0.495). Finally, the COWAT lexicalㅇ was positively correlated with the right middle temporal gyrus (r = 0.569).

No significant correlation was observed between the global network measures and word-list learning memory. We determined that significant correlations existed with the nodal network measures in the LLD-MCI-A(−) group, but we did not find this in the LLD-MCI-A(+) group. The word-list learning score and its z-score showed significant correlations in the LLD-MCI-A(−) group. Word-list learning was negatively correlated with the clustering coefficient of the left middle occipital node (r = − 0.533). Meanwhile, the word-list learning z-score was positively correlated with the nodal strength of the right inferior orbitofrontal cortex (r = 0.517) but was negatively correlated with the clustering coefficient of the left middle occipital node (r = − 0.541).

Regarding the recall and recognition memory domain, all of the significant correlations with the global network measures and the nodal network measures were found in the LLD-MCI-A(+) group, while there was no significant correlation in the LLD-MCI-A(−) group. For the global network measures, the correlations with total strength and the following were positively associated: word-list recall (r = 0.395), word-list recall z-score (r = 0.375), word-list recognition (r = 0.366), and word-list recognition z-score (r = 0.370). Conversely, all of the correlations with the CPL and the following scores were negatively associated: word-list recall (r = − 0.363), word-list recall z-score (r = − 0.319), word-list recognition (r = − 0.429), and word-list recognition z-score (r = − 0.407). For the nodal network measures, all of the correlations were positively associated. The word-list recall and z-scores were positively correlated with the right middle cingulum’s regional efficiency in the LLD-MCI-A(+) group (r = 0.498 and r = 0.477, respectively).

For the visuospatial domain, most of the significant correlations with the global network measures were found in the LLD-MCI-A(+) group. The constructional praxis was negatively correlated with total strength (r = − 0.343), clustering coefficient (r = − 0.369), and small-worldness (r = − 0.353) in the LLD-MCI-A(+) group but was negatively correlated with CPL (r = − 0.305) in the LLD-MCI-A(−) group. The constructional praxis z-score showed significant correlations only in the LLD-MCI-A(+) group. Specifically, it was negatively correlated with total strength (r = − 0.465), clustering coefficient (r = − 0.307), and small-worldness (r = − 0.378), but was positively correlated with CPL (r = 0.413). The constructional praxis recall was positively correlated with the right superior orbitofrontal cortex’s regional efficiency in the LLD-MCI-A(−) group (r = 0.495).

**Figure 2:**
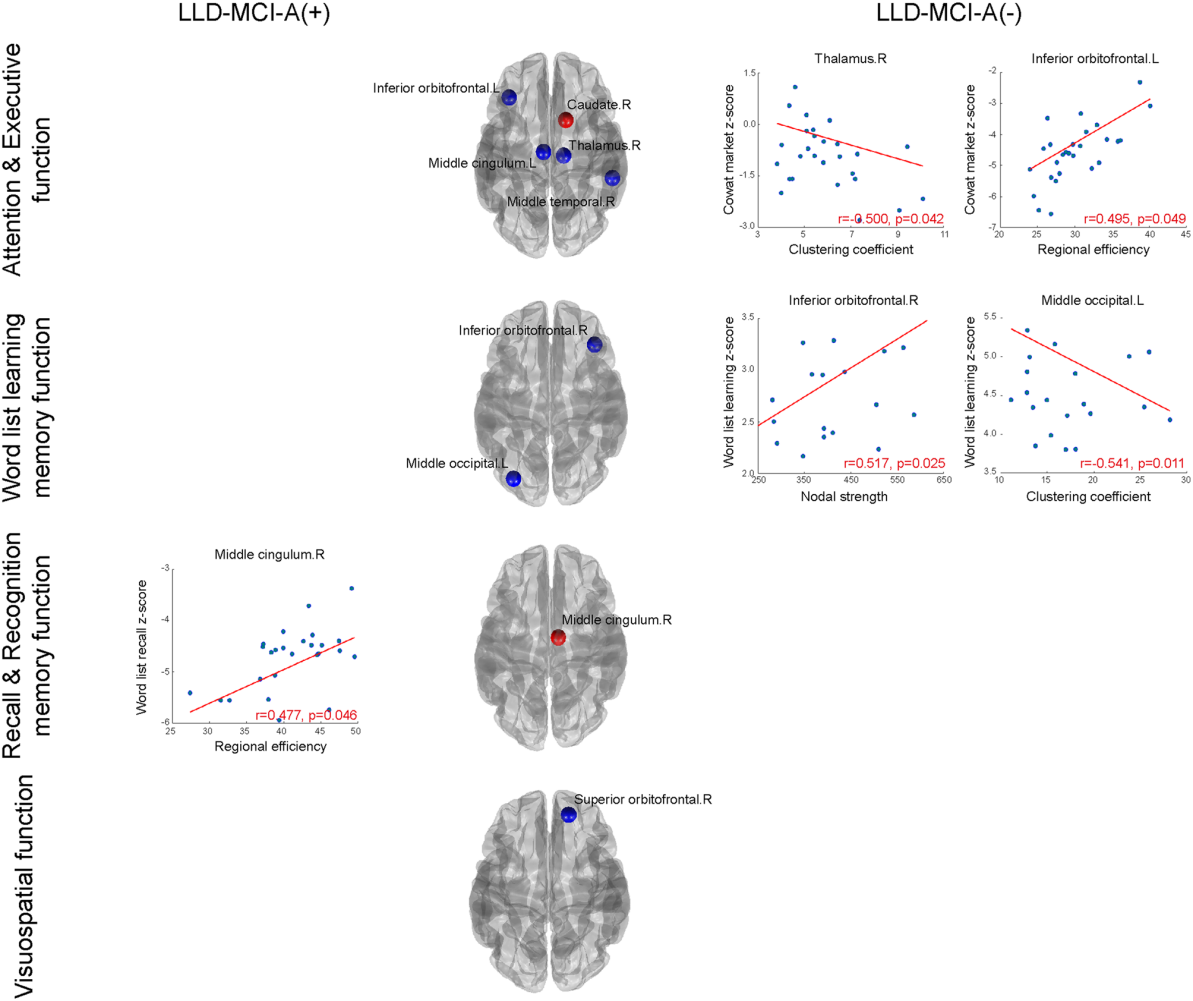
Correlations between the nodal measures and cognitive domains (z-score). Left and right columns show scatter plots of the cognitive domains (z-score) over network measures in the LLD-MCI-A(+) group (leftmost column) and LLD-MCI-A(−) group (rightmost column). Values of the cognitive domains were adjusted, controlling for age, gender, and education level. Lines represent their fitting lines. We denoted partial correlation coefficients and their P-values, controlling for age, gender, and education level. The middle column shows the brain regions associated with significant correlation results, where blue circles represent results from the LLD-MCI-A(−) group and red circles represent results from the LLD-MCI-A(+) group, respectively.

In summary, we found a significant correlation in the global network measures mostly only with attention in the LLD-MCI-A(−) group and with visuospatial domain, recall, and recognition in the LLD-MCI-A(+) group. In contrast, regarding nodal network measures, we found significant correlations mostly with attention, memory, and visuospatial domain in the LLD-MCI-A(−) group and recall and recognition in the LLD-MCI-A(+) group (Fig. 2).

## Discussion

We investigated whether brain amyloid accumulation influences the structural connections linking neuronal units, and this alteration in the structural connections is associated with the distinct cognitive dysfunction in older adults with concomitant depression and MCI. Using a whole-brain connectivity analysis, we found that distinct alterations in whole-brain connectivity may be induced by the presence or absence of brain amyloid accumulation among LLD patients with concomitant MCI. Different impacts of amyloid accumulation on brain network organizations are manifested by the altered nodal strength in the left calcarine and clustering coefficient in the left pallidum in the network. Additionally, the nodal strength of the right inferior orbitofrontal cortex was different in both patient groups from the HOA group. These regions are known to be related to AD and major depression. The calcarine is involved in visual processing and is also associated with learning and recognition^11^. Another report suggests that an AD-related neurodegeneration pattern is apparent in the calcarine along with a visual field map change, although atrophy due to normal aging is also common in this area^12,13^. The orbitofrontal cortex and pallidum play important roles as neuronal circuits that regulate emotion, motivation, and reward and underlie the development of depressive symptoms^14–16^. Our findings suggest that the presence or absence of amyloid accumulation may influence local segregation and transmission of information across the brain network and consequently formulate distinct brain organizations^17^ according to the underlying neuropathology in depressed older adults with cognitive dysfunction.

The relationship between global network properties and cognitive dysfunction reveals how the presence or absence of amyloid accumulation influences specific cognitive domains via distinct alteration of network topological measures with cerebral amyloidopathy. We compared topological properties such as total strength, edge density, small-worldness, CPL, and clustering coefficient between the groups. In the LLD-MCI-A(+) group, word-list recall, recognition, and visuospatial praxis showed significant correlations with total strength and the CPL. This suggests that a disconnect in the entire network influences memory dysfunction in prodromal AD, consistent with our previous study^5^. Additionally, the areas connected with the altered network nodes may disturb the integration of visual information processing, as shown by the significant association between visuospatial praxis and reduced small-worldness^17,18^. The LLD-MCI-A(−) group displayed poor performance during attention tasks associated with alterations of network properties such as diminished total strength and edge density, longer CPL, and reduced small-worldness. These results suggest that the clinical phenotype presenting both LLD and MCI could be subdivided based on the different brain networks provoked by the presence or absence of brain amyloid accumulation. We believe that the alteration in global network measures is linked to cognitive impairment, and the relationships between them are characterized by the underlying neuropathology and neuronal circuit dysfunction. Pathologic amyloid protein begins to accumulate several decades before dementia in AD^19^, so its neurotoxic and neuroinflammatory effects on the brain network possibly result in depressive and cognitive symptoms during the prodromal phase of AD.

The structural connection of inter-regional pathways sheds more insight regarding the differences in the network characterization between groups. The nodal network properties analysis indicated that patients with LLD-MCI-A(+) had a significant correlation between poor word-list recall and regional efficiency in the right middle cingulum. The cingulum is connected to the hippocampus, takes memory information, and integrates it with other important parts of the brain^20^. As shown in our results, regional efficiency in the right middle cingulum node was correlated with recall function in the LLD-MCI-A(+) group. Regional efficiency reflects how well the information propagates across nodes^21^. Previous studies have indicated that regional efficiency might have a biological meaning and serve as a potential marker to predict the risk of AD^22,23^. This suggests that changes in network properties might be early signs of structural impairment caused by amyloid accumulation.

However, in the LLD-MCI-A(−) group, the relationships between memory function and nodal network properties seemed qualitatively different. Their word-list score was correlated with nodal strength in the right inferior orbitofrontal cortex and clustering coefficient in the left middle occipital node. These may be the result of impairments in information traffic flow and segregation within the network, respectively. The orbitofrontal cortex, which is part of the major circuits of depression^16^, is involved in receiving information from the cortical and subcortical structures and in mediating cognitive flexibility^24,25^. In line with our results, some studies have also reported reduced activation of the occipital lobe in depressed patients with cognitive dysfunction. One report suggested that a lower density of gamma-aminobutyric acid (GABA) neurons in the occipital lobe contribute to a low GABA level and an imbalance in neurotransmitters in patients with depression^26^. Another study that used functional magnetic resonance imaging (MRI) found that reduced activation of the occipital lobe may initiate cognitive dysfunction in patients with depression^27^.

Besides memory, inattention and executive function showed significant associations with certain nodal network measurements, clustering coefficient in the right caudate and thalamus, and regional efficiency in the left middle cingulum and left inferior orbitofrontal cortex in the LLD-MCI-A(−) group. These areas may play a role in the manifestation of various depressive symptoms. The limbic–cortical–striatal–pallidal–thalamic circuits are known to be responsible for emotion regulation and are formed by connections between the orbitoprefrontal cortex, amygdala, hippocampal subiculum, striatum, thalamic nuclei, and pallidum^28,29^. Microstructural alterations of the connections within this circuit may lead to loosening of the local group cohesiveness and disturbance of information propagation between nodes.

In summary, amyloid plaque may cause an alteration in brain network topology that leads to distinct cognitive dysfunction in patients with LLD-MCI-A(+). Such susceptible brain networks are also influenced by dysfunction of the fronto-limbic circuits related to depression^29,30^. This may further accelerate the conversion from prodromal AD to dementia^31^. Also, brain network alterations due to amyloid accumulation may contribute to depression and cognitive decline^32^. In contrast, in patients with LLD-MCI-A(−), the brain network alterations may be related to a modified linkage between the fronto-limbic circuits and associated areas^29,30^ and are possibly mediated by the neurotoxic effects of elevated cortisol and reduced brain-derived neurotrophic factor levels^33^. Some of these changes may be state-dependent, and the integrity of the brain network may be partially recovered if optimal therapeutic interventions are applied before depression becomes long-lasting or recurs several times.

This study has some significant clinical implications. Timely identification of AD pathology among older adults with LLD by observing the brain network measures might enable appropriate treatment to be started earlier and improve the prognosis. Unlike prodromal AD patients without depression, it is hard to identify prodromal AD patients with depression among older adults with LLD. Therefore, it may be of great advantage to subdivide patients with LLD and MCI into discrete categories. Observing the integrity of brain connectivity in the early stage may also help predict therapeutic responses and identify novel therapeutic targets.

Investigating the disrupted network organization may also be beneficial by complementing the current limitation of brain amyloid positron emission tomography (PET) imaging. Nowadays, by virtue of molecular imaging techniques, it has become easier to determine prodromal AD than before the emergence of such approaches^34^. The tracers used during brain PET detect amyloid plaque and neurofibrillary tangles, which are key neuropathologies of AD that begin to accumulate 10–15 years before the appearance of AD symptoms^34–36^. Nevertheless, its high procedural cost and radiation exposure limit its wider clinical application. Thus, with the recognition of the significance of early detection in AD and the limitation of current approaches, we should search for an alternative neuroimaging method that may help better identification of neuropathology in older adults with depression and cognitive dysfunction. We believe that MRI, including DWI and its topological network analysis, may shed light on this issue.

This study has several limitations. First, the HOA group was not matched according to age, gender, or education level with the patient groups. We used an ANCOVA and partial correlation coefficients for correcting this effect. Second, the current study employed DWI and thus inherits all of this approach's limitations. We employed ODF reconstruction and high angular resolution diffusion imaging (HARDI) tractography to overcome this, which may better model the crossing fibers. Third, even though we investigated difference in correlation coefficients between patient groups (Table S7 and S8), most of the results did not reach statistical significance due to the small sample size. Thus, all the correlation results contrasting groups should be interpreted with caution.

In conclusion, brain networks using DWI in depressed older patients with comorbid cognitive impairment can distinguish the presence of AD-related neuropathology and explain how brain amyloid accumulation contributes to concomitant cognitive impairment. Alterations in brain network topology reflect different impacts of poor performance on specific cognitive domains based on the presence of amyloid accumulation. Disruptions to network connectivity and integrity caused by neurodegeneration are closely linked to poor recall and recognition in patients with LLD-MCI-A(+). However, inattention and executive function are more influenced by altered networks related to fronto-limbic circuitry dysfunction in patients with LLD-MCI-A(−). This study's findings may help clinicians better predict the prognosis of older adults with depression and guide the planning of tailored interventions.

## Methods

### Participants

Patients were diagnosed with depression and were recruited if they met the criteria for MCI. Depression was diagnosed by two geriatric psychiatrists based on the *Diagnostic and Statistical Manual for Mental Disorders* (DSM-5)^37^. We included older adults with major depressive disorder or subthreshold depression. The research criterion for the diagnosis of subthreshold depression is a depressive episode with insufficient symptoms classified as “other specified depressive disorder” in the DSM-5. MCI was also diagnosed by two geriatric psychiatrists based on the revised diagnostic criteria for MCI proposed by the International Working Group on MCI^38^. Eligible subjects met the following criteria: (1) memory complaints made by the participant or family; (2) objective impairments on neurocognitive tests as indicated by scores with − 1.5 standard deviations below the mean scores of age-, sex- and education-matched healthy older adults (normative data); and (3) no dementia. Neurocognitive tests, used to determine the MCI criteria, included constructional praxis, word-list memory, word-list recall, word-list recognition, constructional recall, verbal fluency, Boston naming test, trail-making test A, digit span forward, and digit span backward^39,40^. Subjective depressive symptoms were also assessed using the Geriatric Depression Scale^41^.

We recruited 74 subjects from Korea University Guro Hospital. Twenty-six patients with amyloid accumulation were included in the LLD-MCI-A(+) group, and 27 patients without amyloid accumulation were included in the LLD-MCI-A(−) group. We also recruited 21 HOA who scored > − 1.5 SD on every cognitive task and had no depression. In our previous study, we reported the findings of fluorodeoxyglucose PET images from 16 subjects with LLD-MCI-A(+), 15 subjects with LLD-MCI-A(−) and 21 HOA^5^. Among them, 51 subjects included in this study. Fifty-three subjects with LLD and MCI in this study also overlapped with subjects of another our study that reported the differences of neuropsychological between 45 LLD-MCI-A(+) and 42 LLD-MCI-A(−) subjects^3^. All subjects were recruited on a voluntary basis. This study was approved by the institutional review board of Korea University Guro Hospital. All methods were performed in accordance with the relevant guidelines and regulations of the ethics committee. Informed consent was obtained from every participant, in accordance with the Declaration of Helsinki.

### Neurocognitive assessment

Cognition was assessed using the Korean version of the Consortium to Establish a Registry for Alzheimer’s Disease assessment packet^42^. The frontal lobe functions, including attention and executive function, were tested using verbal fluency, digit span forward, digit span backward, trail-making test A and abstract reasoning^40,42^. Visuospatial and language functions were tested using the constructional praxis and Boston naming test, respectively^42^. The subtests for memory function included word-list memory, word-list recall, word-list recognition, and constructional recall^42^. The z-score on each neuropsychological test was calculated from age‐, sex‐, and education‐adjusted norms.

### Image acquisition

MRI data were acquired using a 3.0-T MRI (Siemens Trio Trim scanner) at Korea University Guro Hospital. T1-weighted images were acquired using a magnetization-prepared rapid gradient-echo sequence (TE/TR/TI = 2.32 ms/2.3 s/900 ms; 256 × 256 × 192 matrix for 0.9 mm isovoxels). Multiple DWIs were obtained with a standard single-shot, SE-EPI sequence with eddy current–balanced diffusion-weighting gradient pulses. Two sets of DWIs were collected with 22 additional T2-weighted images, where a single set of DWI consisted of a reference volume and 64 volumes with a gradient direction. The parameters of this imaging protocol were: b = 1000 s/mm^2^, TE/TR = 100 ms/3.6 s; matrix = 112 × 112 on 230- × 230-mm field of view; 112 × 112 × 75 matrix for 2 mm isovoxels. Adequate signal-to‐noise ratios were provided by the average of the four magnitudes.

### Amyloid accumulation

Two nuclear medicine specialists who were blinded to the clinical diagnosis and all other clinical findings visually assessed each florbetaben‐PET image based on the regional cortical tracer uptake (RCTU) and brain amyloid plaque load (BAPL) scoring system. The RCTU system grades the tracer uptake (1 = no binding, 2 = minor binding, 3 = pronounced binding) in the lateral temporal cortex, frontal cortex, posterior cingulate cortex/precuneus, or parietal cortex. Each region’s score is condensed into a single three‐grade BAPL scoring system: 1 = no amyloid load, 2 = minor amyloid load, and 3 = significant amyloid load. BAPL scores of “2” and “3” are classified as “amyloid‐positive,” and the BAPL score of “1” is regarded as “amyloid‐negative”^43^. Older adults with depression who had a BAPL score of 2 or 3 were categorized into the amyloid-positive group, while those with a score of 1 were placed into the amyloid-negative group. All subjects of HOA group also had a BALP score of 1.

### Network construction

The brain network consists of nodes, anatomically defined brain regions, edges, and connections between any of these. We included 78 cortical and 12 subcortical brain regions as the nodes, which are defined in the automated anatomical labeling atlas (AAL)^44^. To delineate them in each subject’s diffusion space, we co-registered the DWI with the T1-weighted image and nonlinearly registered the T1-weighted image with the standard Montreal Neurological Institute template using FSL Toolkit (version 5.0.9)^45^.

We used whole-brain tractography using the processed DWIs through the diffusion toolkit and TrackVis (version 0.6.0.1)^46,47^ to estimate the strength of the edges. First, the eddy toolbox of FSL’s Diffusion Toolkit (version 3.0) was performed to register all volumes with the gradient direction of DWIs to their reference volume of DWIs^48^. We employed HARDI tractography since it may represent crossing fibers better^49,50^. Although our DWIs were not acquired using the HARDI MRI protocol since they have many diffusion directions, we could apply HARDI tractography^51^. We note that we restricted the seed regions as the white matter to avoid artifacts in the tractography. Finally, we obtained structural connectivity matrices from the defined nodes and tractography by counting the number of streamlines between any pair of nodes using the University of California, Los Angeles multimodal connectivity package (http://ccn.ucla.edu/wiki/index.php). The number of streamlines may be considered as the projection strength of the white matter pathways^52^. The detailed procedure is shown in the supplementary material.

### Network measures

We computed the network measures using the Brain Connectivity Toolbox (https://sites.google.com/site/bctnet/) to quantify the global and local properties of the network^17^. We measured the nodal degree, nodal strength, nodal clustering coefficients, and regional efficiency for the nodal level, edge density, total strength, clustering coefficient, CPL, and small-worldness for the global level. The nodal degree is the number of neighboring nodes linked to a node, while the nodal strength is the sum of edge weights linked to the node. They capture the centrality of the node and estimate the direct influence on its neighbors. The edge density of a network captures the number of all existing connections, while its total strength is the sum of all its edge weights. They may be affected by the overall deterioration of the white matter due to neurodegeneration; white matter deterioration may impair the integrity of white matter tracts, weakening edge weights, and even disconnecting edges. The nodal clustering coefficient of a node measures the level of local clustering of its neighborhood, and the clustering coefficient of a network is the average of the values in the network. The clustering coefficient is used to measure small-worldness, combined with CPL, which measures global integration. Specifically, CPL is the average of the shortest path lengths between all pairs of nodes in the network. A shorter path length represents more efficient communication between nodes, and therefore, a lower CPL reflects better overall global integration. The small world characteristics capture the balance between good local communication measured by the high clustering coefficient and good global communication measured by the short CPL. Thus, it is defined by the ratio of the clustering coefficient to CPL. Similar to CPL, regional efficiency measures the level of communication excellence based on the shortest path lengths, but it measures that of a certain node, defined by the average of the shortest path lengths from the node to all the others. We used the MATLAB brain connectivity toolbox to compute these measures^17^. A more formal description of the network measures is shown in the supplementary materials.

### Group comparison

We primarily compared the topological network measures of each patient group with the HOAs to identify the degree of its disintegration. We conducted multiple-comparison correction using permutation testing^53^ and the FDR procedure^54^ to identify differences in the network measures between groups. We first tested the difference between the three groups by permutation-based ANCOVA^20^, controlling for the effects of age, gender, and education level. Then, we performed a permutation-based ANCOVA for three pairs of groups and corrected across three pairwise comparisons through the FDR procedure for post-hoc tests. For the nodal measures, we also performed the FDR procedure across 90 nodes. We used our in-house codes for permutation-based ANCOVA under MATLAB R2017a (The MathWorks Inc, Natick, MA, USA), where the number of permutations is 10,000.

### Correlation analysis

We conducted a correlation analysis involving the network measures and neurocognitive tests, controlling for age, gender, and education level by computing Pearson’s partial correlation coefficients in each MCI group separately^55^. The cognitive scores of the Consortium to Establish a Registry for AD^39^ and the Seoul Neuropsychological Screening Battery were used^40^. We divided the cognitive domains into the following groups: attention, executive function, memory, and visuospatial function. A list of the neurocognitive tests used is presented in Tables 2 and 3.

## Supplementary Information

Supplementary Information.
