# Supplementary material for: Brain network analysis reveals that amyloidopathy affects comorbid cognitive dysfunction in older adults with depression
Source: Sci Rep. 2021 Feb 22;11:4299. doi: 10.1038/s41598-021-83739-3 (PMC7900108; doi:10.1038/s41598-021-83739-3)
Supplement: Supplementary file 1 — Supplementary Information. [file 41598_2021_83739_MOESM1_ESM.docx]

**Brain network analysis reveals that amyloidopathy affects comorbid cognitive dysfunction in older adults with depression**

Suji Lee^1,^†, Daegyeom Kim^2,^†, HyunChul Youn^3^, Won Seok William Hyung^4^, Sangil Suh^5^, Marcus Kaiser^6,7,8,9^, Cheol E. Han^2,10*^, Hyun-Ghang Jeong^1,4,*^

^1^ Department of Biomedical Sciences, Korea University Graduate School, Seoul, Republic of Korea

^2^Department of Electronics and Information Engineering, Korea University, Sejong, Republic of Korea

^3^Department of Psychiatry, Soonchunhyang University Bucheon Hospital, Bucheon, Republic of Korea

^4^Department of Psychiatry, Korea University Guro Hospital, Korea University College of Medicine, Seoul, Republic of Korea

^5^Department of Radiology, Korea University Guro Hospital, Korea University College of Medicine, Seoul, Republic of Korea

^6^Interdisciplinary Computing and Complex BioSystems (ICOS) research group, School of Computing, Newcastle University, Newcastle upon Tyne NE4 5TG, United Kingdom

^7^Institute of Neuroscience, Newcastle University, the Henry Wellcome Building, Newcastle upon Tyne NE2 4HH, United Kingdom

^8^Department of Functional Neurosurgery, Ruijin Hospital, School of Medicine, Shanghai Jiao Tong University, Shanghai 200025, China

^9^Precision Imaging Beacon, School of Medicine, University of Nottingham, Nottingham, NG7 2UH

^10^Interdisciplinary Graduate Program for Artificial Intelligence Smart Convergence Technology, Korea University, Sejong, Republic of Korea

†The first two authors contributed equally to this article.

*Corresponding: [cheolhan@korea.ac.kr](mailto:cheolhan@korea.ac.kr) (C.E. Han); [jeonghg@korea.ac.kr](mailto:jeonghg@korea.ac.kr) (H.-G. Jeong)

*Supplementary Information*

**Network Construction**

The brain network consists of nodes, anatomically defined brain regions, edges, and connections between these. We defined the nodes based on the T1-weighted magnetic resonance (MR) images and estimated the edge strength based on the diffusion-weighted images (DWIs).

We included 78 cortical and 12 subcortical brain regions as the nodes defined in the automated anatomical labeling (AAL) ^1^. To delineate them in each subject's diffusion space, we co-registered the DWI with the T1-weighted image and nonlinearly registered the T1-weighted image with the standard Montreal Neurological Institute (MNI) template. The overall registration procedure is shown in Figure S1. We first extracted the brain of each subject on the MRI scans by removing non-brain tissue using the brain extraction tool (version 2.1) of the FSL Toolkit ^2^. Then, we performed coregistration between the DWIs and T1-weighted images of each subject using the affine registration method (FSL's linear registration toolbox version 6.0, rigid-body) ^2^. We nonlinearly registered the brain on the T1-weighted image using the standard MNI brain template and FSL's nonlinear registration toolbox (version 6.0) ^2^. We transformed the regions of interest (ROIs, i.e., brain regions) defined in the AAL atlas into each subject's diffusion space by inversely applying the registration parameters. We excluded the ROIs in the cerebellum, and thus, the number of ROIs achieved was 90 (including 78 cortical and 12 subcortical brain regions).

**
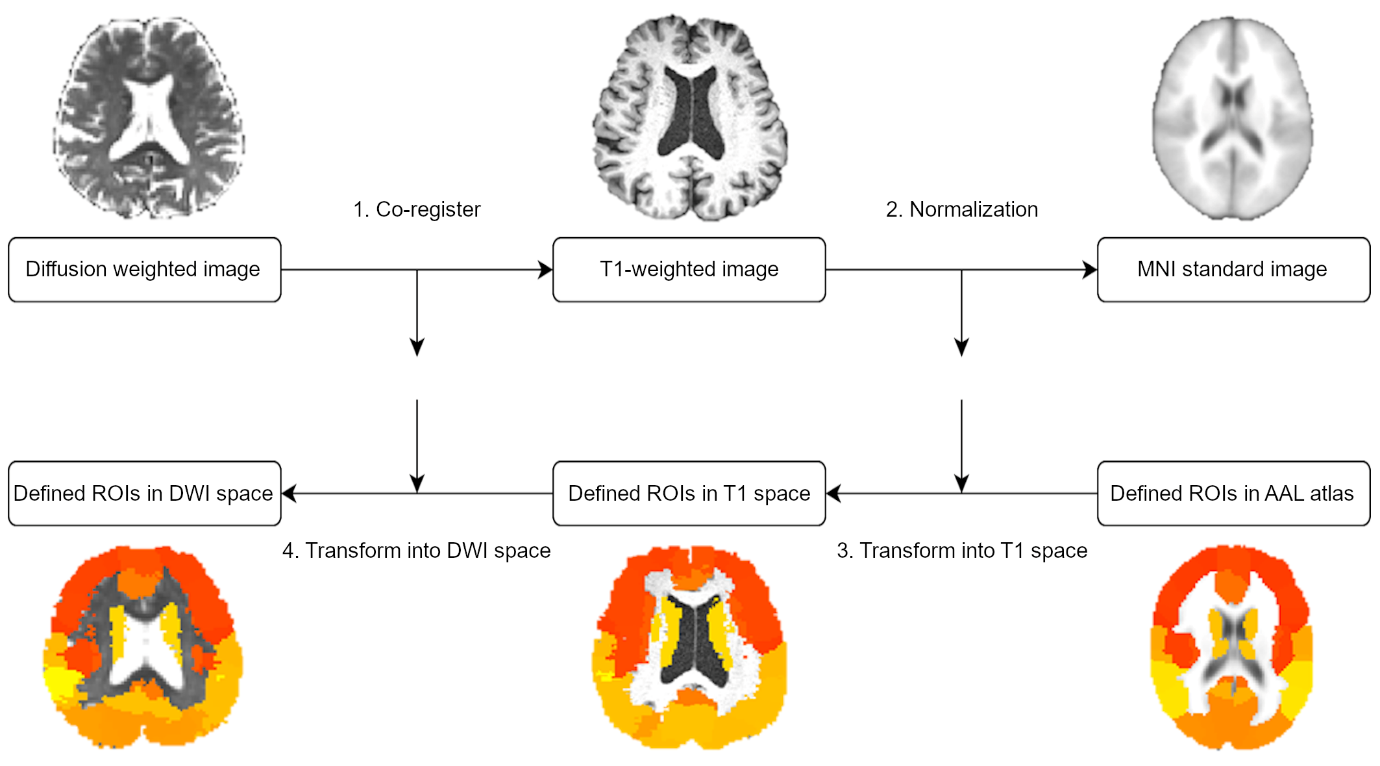
**

**Supplemental Figure S1. Overall procedure to define the nodes using AAL atlas.**

We used whole-brain tractography and the processed DWIs to estimate the strength of the edges. Before performing the tractography, we first merged the DWIs because they were acquired twice in each subject using the FSL Toolkit ^3^ (version 5.0.9, Analysis Group, FMRIB, Oxford, UK). We divided the DWI volumes into three: two sets of DWIs that consisted of a reference volume and 64 volumes with gradient directions, and one set that contained 22 additional T2 volumes without a gradient direction. We separately performed the eddy toolbox of FSL's Diffusion Toolkit (version 3.0) for the two sets of DWIs by registering all volumes with the gradient direction to their own reference volume ^4^. By registering the reference volumes in this manner, we aligned the two sets of eddy current–corrected DWIs. We appropriately rotated the gradient direction vectors during the alignment procedure. The two reference volumes and the additional T2 volumes without a gradient direction were averaged, resulting in a single reference volume.

We performed high angular resolution diffusion imaging (HARDI) tractography to obtain streamline tractography from the processed DWIs since it may better represent crossing fibers ^5,6^. To perform HARDI tractography, we first reconstructed the orientation density function (ODF) from the DWI, where ODF models radially projected the diffusion direction through the HARDI gradient matrix using the diffusion toolkit along with TackVis (version 0.6.0.1) ^7,8^. Specifically, we computed ODFs for each voxel of the DWIs using the normalized and dimensionless ODF estimator, similar to the process used when estimating a tensor for each voxel for diffusion tensor imaging ^9^. Since ODF represents the voxel's diffusion direction more precisely than the tensor, which only captures one primary direction, HARDI tractography may help to resolve the issue of crossing fibers. Using the reconstructed ODF, we performed whole-brain tractography using the Fiber Assignment by Continuous Tracking algorithm with an angular threshold of 45˚ through the diffusion toolkit ^7,8^. We note that we restricted the seed regions of the tractography as the brain mask that only contains the white matter to avoid artifacts in the tractography. We extracted such masks using FSL's automated segmentation tool ^10^.

Finally, we obtained connectivity matrices from the defined and registered ROIs and tractography results by counting the number of streamlines between any pair of ROIs using the University of California, Los Angeles multimodal connectivity package (<http://ccn.ucla.edu/wiki/index.php>), where the nodes are anatomically defined ROIs. The edge weights are the number of streamlines between any pair of ROIs. As a result, we obtained a 90-by-90 connectivity matrix, given that we used 90 brain regions as nodes (i.e., 78 cortical and 12 subcortical brain regions).

**Network Measures**

We computed network measures using the brain connectivity toolbox (https://sites.google.com/site/bctnet/) to quantify the global and local properties of the network ^11^. We measured the nodal degree, total strength, nodal strength, edge density, regional efficiency, clustering coefficient, characteristic path length (CPL), and small-worldness. We used the MATLAB implementation of the brain connectivity toolbox to compute those measures ^11^.

**Degree.** The degree of a node is the number of its neighboring nodes connected to the node. Specifically, the degree of the ith node, *K_i_*, is computed by summing $a_{ij}$ from the node in Equation 1, where $a_{ij}=1$ if a connection between the ith and jth node exists; otherwise, $a_{ij}=0$. *N* is the number of nodes in the network. The greater the degree of a node is, the more edges the node is connected with.

$K_{i}=\sum_{j\in N} a_{ij}$ (1)

**Strength.** Strength represents the sum of all edge weights. The nodal strength of the ith node, $S_{i}$, is calculated by the summation of all edge weights connected with the node (Equation 2). Similar to the nodal degree, where *w_ij_* is the edge weight between the ith and jth node and *N* is the number of nodes, summing *w_ij_* from the ith node results in the nodal strength of the node. The nodal strength indicates how strongly the node is connected with its neighboring nodes.

$S_{i}=\sum_{j\in N} w_{ij}$ (2)

While the nodal strength is calculated for a certain node, the total strength is calculated for the whole network by the summation of all edge weights. Since the connectivity matrix is symmetric, the total strength is one-half of the summation (Equation 3). The total strength refers to how well all of the nodes are connected.

$S=\frac{\sum_{i\in N} \sum_{j\in N} w_{ij}}{2}$ (3)

**Density.** Density is the fraction of the number of existing connections to the number of all possible connections in a network. The number of existing connections is indicated by *E*. The number of possible connections for *N* nodes is *N(N-1)/2* since the connectivity matrix is symmetric, where the number of all nodes is *N* (Equation 4).

$\rho=\frac{E}{N(N-1)/2}$ (4)

**Clustering Coefficient.** The local clustering coefficient of a node is the fraction of the number of triangles connected with the node over the number of possible connections between all neighboring nodes connected with the node ^12^. In the case of a weighted clustering coefficient, the number of triangles is replaced by the total intensity of triangles. If the given node is denoted by i and its two neighboring nodes are denoted by j and k, $w_{ij}$, $w_{jk}$, and $w_{ki}$ represent edge weights between any pair of two nodes, respectively. The total intensity of triangles attached to the ith node is given by Equation 5. That is the summation of the cubic-root of products of all edge weights for all connected triangles.

$t_{i}=\sum_{j,k\in N} (w_{ij}w_{jk}w_{ki})^{1/3}$ (5)

The nodal clustering coefficient is defined using the average intensity of the triangles around a node. Since the number of possible connections is$K_{i}(K_{i}-1)/2$, where $K_{i}$ is the degree of the ith node, the nodal clustering coefficients of the ith node can be written as in Equation 6.

$C_{i}=\frac{2}{K_{i}(K_{i}-1)}\sum_{j,k\in N} t_{i}$ (6)

The clustering coefficient of the whole network, *C,* is defined as the average of the local clustering coefficients over all nodes (Equation 7). It indicates how well all nodes in the network are locally clustered.

$C=\frac{\sum_{i\in N} C_{i}}{N}$ (7)

**CPL.** CPL is the average of the shortest path lengths between all node pairs in the network ^13,14^. The shortest path length is the minimum distance between a pair of nodes and reciprocally represents the level of efficient communication of information between two nodes. A shorter CPL represents a more efficient level of information communication in the brain.

Specifically, we computed the shortest path length, $d_{ij}$, which is defined as the minimum distance between the ith and jth nodes. Before the computation, we performed mapping from weight to distance, i.e., the distance is the reciprocal of the edge weights between two nodes since a large edge weight should be interpreted as a shorter distance.

If there is no possible route between the ith and jth nodes, then the shortest path length is infinity. In this case, CPL, the average of all of the shortest paths, also becomes infinity. To prevent this, we excluded the infinite-valued short paths during averaging (see Equation 8 where D is the number of finites $d_{ij}$).

$L=\frac{\sum_{d_{ij}\neq\infty} d_{ij}}{D}$ (8)

**Small-Worldness.** The small-world network proposed by Watts and Strogatz ^13^ is interposed between the regular and random networks. It has as high a clustering coefficient as the regular network does and as low a CPL as the random network does. A high clustering coefficient indicates the local clustering of nodes (i.e., efficient communication between local nodes), while a low CPL in the brain network indicates an efficient exchange of information across the network (i.e., global integration of information). Thus, smallworldness, $\sigma$, shows the balance between the local segregation and global integration of the brain networks. It is computed as the ratio between the clustering coefficient and CPL (Equation 9).

$\sigma=\frac{C}{L}$ (9)

**Regional Efficiency.** The regional efficiency of the ith node is the reciprocal of the harmonic mean of the shortest path lengths between this node and all other nodes in the network ^15,16^ (Equation 10). Since the reciprocal of the shortest path length suggests the efficiency of information transfer between two nodes, the average of the reciprocal of the shortest path lengths from the ith node to all other nodes indicates how efficiently the node is connected with the network on average.

$regionalefficiency_{i}=\frac{1}{N -1}\sum_{i\neq j\in N} \frac{1}{d_{ij}}$ (10)

**Permutation-based analysis of covariance (ANCOVA)**

For the group comparison, we used the permutation-based analysis of covariance (ANCOVA) ^17^. We could not use simple permutation testing for the group mean difference since we aimed to compare the measures of three groups: HOA, LLD-MCI-A(+), and LLD-MCI-A(-). Moreover, we needed to control for covariates for a more accurate and fair comparison, including age, gender, and education level. Specifically, we resampled the dataset *N* times using random permutation of all subjects by keeping the number of subjects in each group, where *N* is the number of permutations. We computed test statistics *F* for the original and permuted assignments using an ANCOVA, resulting in a null distribution for the resampled dataset. Finally, we obtained the significance level by counting the number of samples whose test statistics were larger than those from the original assignment. For computing the test statistics, we used our in-house codes and LinStat library (2006b) ^18^ under MATLAB (2017a; MathWorks, Natick, MA, USA).

**Comparison of correlation coefficients**

We performed the correlation analysis between the network measures and neurocognitive tests, controlling for age, gender and education level through Pearson’s partial correlation coefficients in each MCI group separately. To investigate whether two correlation coefficients were significantly different, we first converted them into Z-values using Fisher's Z-transformation ^19^ (Equation 11).

$Z= \frac{1}{2}ln\frac{1+r}{1-r}$ (11)

where r is the correlation coefficient. Then, we calculated the observed z statistics using the equation 12, where *Z_1_* and *Z_2_* are the transformed Z-values for two patient groups respectively, and *N_1_* and *N_2_* are their corresponding sample sizes respectively.

$Z_{observed}= \frac{Z_{1}-Z_{2}}{\sqrt{\frac{1}{N_{1}-3}+\frac{1}{N_{2}-3}}}$ (12)

Since the Z-values approximately follows the normal distribution, *Z_observed_* also does, and thus its significance level can be estimated through the normal distribution. As an example, if it is greater than 1.96 which is a critical value at the significance level of 0.05, two group's correlation coefficients are significantly different each other.

We added group comparison results to our correlation analysis. For the correlation between global network measures and cognitive scores (Table 2), we found significant difference in the visuospatial functions (Table S7): constructional praxis and smallworldness (*Z_observed_*=-2.118, p=0.034), constructional praxis z-score and total strength (*Z_observed_*=-2.360, p=0.018), and constructional praxis z-score and smallworldness (*Z_observed_*=-2.036, p=0.042). All other group comparison results did not reach statistical significance (Table S7). We also added group comparison results to the correlation between nodal measures and cognitive scores (Table 3). No group comparison results reached statistical significance (Table S8).

The reason of insignificant group difference in correlation coefficients may be due to the small sample size. The Fisher’s Z transformation itself requires at least a sample size of 25 for accurate transformation ^20^; the size of our data is near the boundary. A previous theoretical analysis noted that a sample size of 100 or more may be required for significant difference on the order of 0.3 when comparing two correlation coefficients ^21^. Thus, this group comparison results should also be interpreted with caution.

**References**

1 Tzourio-Mazoyer, N. *et al.* Automated anatomical labeling of activations in SPM using a macroscopic anatomical parcellation of the MNI MRI single-subject brain. *Neuroimage* **15**, 273-289, doi:10.1006/nimg.2001.0978 (2002).

2 Jenkinson, M., Bannister, P., Brady, M. & Smith, S. Improved optimization for the robust and accurate linear registration and motion correction of brain images. *Neuroimage* **17**, 825-841, doi:10.1016/s1053-8119(02)91132-8 (2002).

3 Jenkinson, M., Beckmann, C. F., Behrens, T. E., Woolrich, M. W. & Smith, S. M. Fsl. *Neuroimage* **62**, 782-790, doi:10.1016/j.neuroimage.2011.09.015 (2012).

4 Andersson, J. L. R. & Sotiropoulos, S. N. An integrated approach to correction for off-resonance effects and subject movement in diffusion MR imaging. *NeuroImage* **125**, 1063-1078, doi:<https://doi.org/10.1016/j.neuroimage.2015.10.019> (2016).

5 Descoteaux, M., Deriche, R., Knösche, T. R. & Anwander, A. Deterministic and probabilistic tractography based on complex fibre orientation distributions. *IEEE Trans Med Imaging* **28**, 269-286, doi:10.1109/tmi.2008.2004424 (2009).

6 Behrens, T. E. *et al.* Characterization and propagation of uncertainty in diffusion-weighted MR imaging. *Magn Reson Med* **50**, 1077-1088, doi:10.1002/mrm.10609 (2003).

7 Mori, S., Crain, B. J., Chacko, V. P. & van Zijl, P. C. Three-dimensional tracking of axonal projections in the brain by magnetic resonance imaging. *Ann Neurol* **45**, 265-269, doi:10.1002/1531-8249(199902)45:2<265::aid-ana21>3.0.co;2-3 (1999).

8 Wang, R., Benner, T., Sorensen, A. G. & Wedeen, V. J. in *Proc Intl Soc Mag Reson Med.* (Berlin).

9 Sotiropoulos, S. N. *et al.* Inference on constant solid angle orientation distribution functions from diffusion-weighted mri. *OHBM. Quebec, Canada* (2011).

10 Zhang, Y., Brady, M. & Smith, S. Segmentation of brain MR images through a hidden Markov random field model and the expectation-maximization algorithm. *IEEE Trans Med Imaging* **20**, 45-57, doi:10.1109/42.906424 (2001).

11 Rubinov, M. & Sporns, O. Complex network measures of brain connectivity: uses and interpretations. *Neuroimage* **52**, 1059-1069, doi:10.1016/j.neuroimage.2009.10.003 (2010).

12 Onnela, J. P., Saramäki, J., Kertész, J. & Kaski, K. Intensity and coherence of motifs in weighted complex networks. *Phys Rev E Stat Nonlin Soft Matter Phys* **71**, 065103, doi:10.1103/PhysRevE.71.065103 (2005).

13 Watts, D. J. & Strogatz, S. H. Collective dynamics of 'small-world' networks. *Nature* **393**, 440-442, doi:10.1038/30918 (1998).

14 Sporns, O. Network analysis, complexity, and brain function. *Complexity* **8**, 56-60 (2002).

15 Hsu, T. W. *et al.* Impaired small-world network efficiency and dynamic functional distribution in patients with cirrhosis. *PLoS One* **7**, e35266, doi:10.1371/journal.pone.0035266 (2012).

16 Latora, V. & Marchiori, M. Efficient behavior of small-world networks. *Phys Rev Lett* **87**, 198701, doi:10.1103/PhysRevLett.87.198701 (2001).

17 Cho, E. B. *et al.* White Matter Network Disruption and Cognitive Dysfunction in Neuromyelitis Optica Spectrum Disorder. *Front Neurol* **9**, 1104, doi:10.3389/fneur.2018.01104 (2018).

18 Mulchrone, K. F. LinStat, a program for calculating finite strain from populations of lines, running simulations and an investigation of error behaviour. *Computers & geosciences* **29**, 639-646 (2003).

19 Fisher, R. A. Frequency Distribution of the Values of the Correlation Coefficient in Samples from an Indefinitely Large Population. *Biometrika* **10**, 507-521, doi:10.2307/2331838 (1915).

20 Daniel, W. W. & Cross, C. L. *Biostatistics: A Foundation for Analysis in the Health Sciences (10th edition)*. 453-455 (Wiley, 2013).

21 Phillips, P. C. Designing Experiments to Maximize the Power of Detecting Correlations. *Evolution* **52**, 251-255, doi:10.1111/j.1558-5646.1998.tb05158.x (1998).

**Supplementary Figure S2. Correlation between age and Mini-Mental State Examination (MMSE), and education level and MMSE**


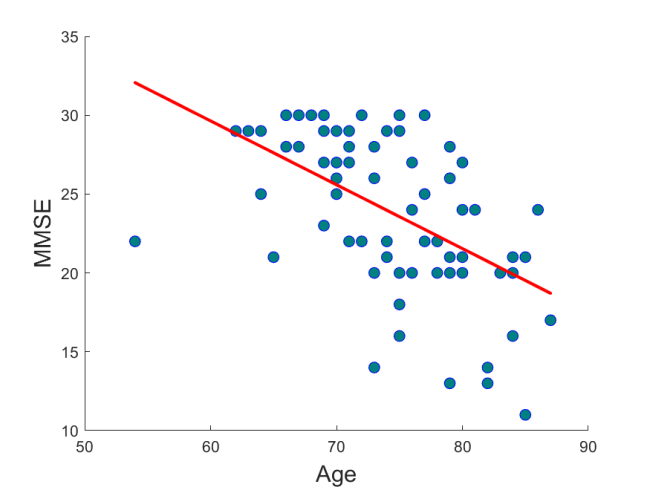

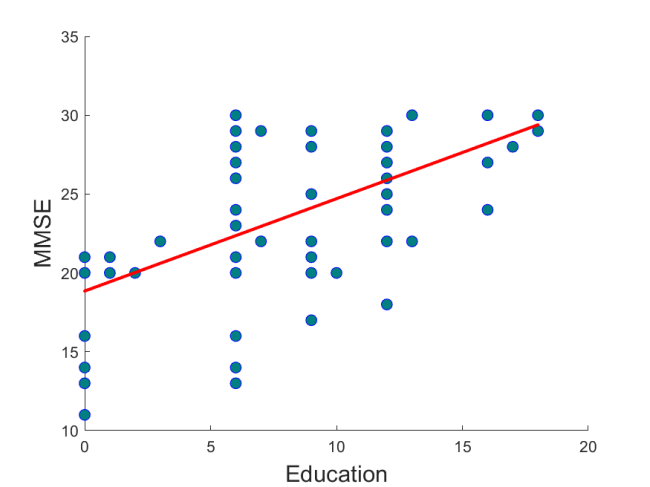


**Supplementary Table S1. Differences in global network measures between groups**

| **Global network measure** | **HOA** | **LLD-MCI-A(+)** | **LLD-MCI-A(-)** | **Three-group comparison** | **HOA**  **vs.**  **LLD-MCI-A(+)** | **HOA**  **vs.**  **LLD-MCI-A(-)** | **LLD-MCI-A(+)**  **vs.**  **LLD-MCI-A(-)** |
| --- | --- | --- | --- | --- | --- | --- | --- |
| **Total strength** | 43373.0340±2739.5093^a^ | 40685.7785±3080.3643 | 39877.9875±2607.6030 | 0.2721^b^ | 0.1044^c^ | 0.5441^c^ | 0.5441^c^ |
| **Edge density** | 0.2160±0.0101 | 0.2119±0.0121 | 0.2110±0.0117 | 0.8481 | 0.6444 | 0.9368 | 0.9368 |
| **Clustering coefficient** | 19.5367±1.2922 | 19.1437±1.4569 | 18.5800±1.1005 | 0.4074 | 0.9791 | 0.9791 | 0.5937 |
| **Characteristic path length** | 0.0166±0.0039 | 0.0201±0.0049 | 0.0204±0.0043 | 0.7688 | 0.2526 | 0.9465 | 0.9465 |
| **Small-worldness** | 660.0403±64.2895 | 619.6482±66.2436 | 602.5364±51.2898 | 0.6812 | 0.7410 | 0.8352 | 0.7410 |

^a^mean±standard deviation, controlling for age, gender, and education level.

^b^P-values from the permutation-based ANCOVA, controlling for age, gender, and education level between the three groups.

^c^P-values from post-hoc tests using three pairwise permutation-based ANCOVAs, controlling for age, gender, and education level; the collected P-values were FDR-adjusted across the three pairwise comparisons.

**Supplementary Table S2. Group differences in nodal network measures (FDR-survived results only)**

| Nodal Network measure | **Nodes** | **HOA** | **LLD-MCI-A(+)** | **LLD-MCI-A(-)** | **Three-group comparison** | **HOA**  **vs.**  **LLD-MCI-A(+)** | **HOA**  **vs.**  **LLD-MCI-A(-)** | **LLD-MCI-A(+)**  **vs.**  **LLD-MCI-A(-)** |
| --- | --- | --- | --- | --- | --- | --- | --- | --- |
| Nodal strength | Left calcarine | 2579.7820±196.4793 ^a^ | 2731.2453±288.6206 | 2442.0984±258.1014 | 0.0090^b,d^ | 0.0324^c^ | 0.6690^c^ | 0.0021^c,d^ |
|  | Right inferior orbitofrontal | 868.0110±125.6880 | 691.9264±119.8962 | 640.1641±102.1215 | 0.0360^b,d^ | 0.0244^c,d^ | 0.0012^c,d^ | 0.1606^c^ |
| Clustering coefficient | Left pallidum | 27.1902±2.9873 | 28.4575±3.7485 | 24.1069±2.7305 | 0.0090^b,d^ | 0.7259^c^ | 0.0711^c^ | 0.0003^c,d^ |

^a^mean±standard deviation, controlling for age, gender, and education level.

^b^P-values from the permutation-based ANCOVA, controlling for age, gender, and education level between the three groups.

^c^P-values from post-hoc tests using three pairwise permutation-based ANCOVAs, controlling for age, gender, and education level; the collected P-values were FDR-adjusted across the three pairwise comparisons.

^d^P-values < 0.05.

**Supplementary Table S3. Group difference of the nodal degree**

| Nodes | HOA | LLD-MCI-A(+) | LLD-MCI-A(-) | Three-group  comparison | HOA  vs.  LLD-MCI-A(+) | HOA  vs.  LLD-MCI-A(-) | LLD-MCI-A(+)  vs.  LLD-MCI-A(-) |
| --- | --- | --- | --- | --- | --- | --- | --- |
| Precentral_L | 23.5296±3.4041^a^ | 23.4529±2.8592 | 23.6982±3.1282 | 0.8329^b^ | 0.7120^c^ | 0.7120^c^ | 0.7120^c^ |
| Precentral_R | 22.2737±3.7644 | 21.2764±2.9202 | 22.4083±2.8339 | 0.7231 | 0.5666 | 0.5666 | 0.3051 |
| Frontal_Sup_L | 29.3364±4.2898 | 30.8709±4.2259 | 29.9250±3.7947 | 0.7231 | 0.6623 | 0.5013 | 0.6623 |
| Frontal_Sup_R | 37.7748±4.7843 | 37.8601±6.4842 | 38.4335±4.8336 | 0.8857 | 0.9821 | 0.9821 | 0.9821 |
| Frontal_Sup_Orb_L | 23.1588±4.7735 | 20.2874±4.2686 | 20.6624±4.5250 | 0.7231 | 0.6876 | 0.6876 | 0.6876 |
| Frontal_Sup_Orb_R | 16.7559±4.5213 | 14.5488±3.9404 | 13.0676±4.1065 | 0.5515 | 0.2448 | 0.1005 | 0.2448 |
| Frontal_Mid_L | 12.1364±3.0503 | 12.8897±3.6355 | 11.9614±3.5517 | 0.7857 | 0.6170 | 0.6170 | 0.6170 |
| Frontal_Mid_R | 24.2421±3.6338 | 24.0989±4.4526 | 23.5633±4.1249 | 0.8658 | 0.9330 | 0.9330 | 0.9330 |
| Frontal_Mid_Orb_L | 16.7944±3.3255 | 15.5142±3.8745 | 14.7482±2.7157 | 0.7231 | 0.7541 | 0.1476 | 0.7541 |
| Frontal_Mid_Orb_R | 14.1425±4.5116 | 12.6046±4.0954 | 9.5525±2.8220 | 0.2250 | 0.5181 | 0.0228 | 0.0171 |
| Frontal_Inf_Oper_L | 10.7253±1.6261 | 9.6764±2.4558 | 10.7813±1.9115 | 0.5515 | 0.3831 | 0.3831 | 0.1326 |
| Frontal_Inf_Oper_R | 7.2348±3.0216 | 5.4756±2.1352 | 6.0429±2.0658 | 0.7231 | 0.3882 | 0.4602 | 0.4602 |
| Frontal_Inf_Tri_L | 26.9926±3.2960 | 24.4316±2.9259 | 25.1047±4.2617 | 0.7231 | 0.5334 | 0.7503 | 0.7425 |
| Frontal_Inf_Tri_R | 15.5271±4.5476 | 12.2498±2.9932 | 12.4076±2.3827 | 0.3369 | 0.0372 | 0.0372 | 0.7507 |
| Frontal_Inf_Orb_L | 38.8487±5.4980 | 36.6723±4.5214 | 36.3293±4.3831 | 0.7857 | 0.8139 | 0.8139 | 0.8139 |
| Frontal_Inf_Orb_R | 23.9104±4.8986 | 18.5768±4.7410 | 17.9919±3.6329 | 0.1800 | 0.0748 | 0.0108 | 0.7302 |
| Rolandic_Oper_L | 15.4177±1.8909 | 16.0006±1.6999 | 16.2982±2.0432 | 0.7231 | 0.4434 | 0.4434 | 0.4434 |
| Rolandic_Oper_R | 11.3776±1.4357 | 11.0082±1.6475 | 10.6904±1.3928 | 0.9012 | 0.8828 | 0.8828 | 0.8828 |
| Supp_Motor_Area_L | 20.0810±3.7593 | 20.4604±3.1950 | 21.7815±2.8654 | 0.5515 | 0.8513 | 0.0075 | 0.2853 |
| Supp_Motor_Area_R | 22.2598±3.3692 | 23.4899±3.3374 | 23.7030±3.6586 | 0.7231 | 0.5959 | 0.5664 | 0.8972 |
| Olfactory_L | 17.3011±4.2410 | 17.6528±4.0350 | 17.7207±3.5606 | 0.9999 | 0.8894 | 0.8894 | 0.8894 |
| Olfactory_R | 13.8924±2.7853 | 13.2464±2.5527 | 11.5949±2.9153 | 0.5515 | 0.6776 | 0.6776 | 0.1305 |
| Frontal_Sup_Medial_L | 31.8144±4.2043 | 28.6799±4.5622 | 30.1944±2.4060 | 0.6785 | 0.2310 | 0.4316 | 0.2310 |
| Frontal_Sup_Medial_R | 29.5732±5.6907 | 30.3961±4.6605 | 31.9343±4.8067 | 0.7231 | 0.7416 | 0.4930 | 0.4930 |
| Frontal_Med_Orb_L | 14.1117±3.0511 | 14.9998±2.6063 | 15.3187±2.6042 | 0.7231 | 0.4630 | 0.4131 | 0.4630 |
| Frontal_Med_Orb_R | 17.7828±2.0676 | 18.9399±3.0835 | 18.0843±3.2770 | 0.7231 | 0.6076 | 0.4787 | 0.4787 |
| Rectus_L | 22.7404±3.0954 | 21.1003±4.9789 | 20.9685±3.3504 | 0.7231 | 0.4653 | 0.7209 | 0.8291 |
| Rectus_R | 22.5870±2.6051 | 22.4433±3.5584 | 21.5064±3.7256 | 0.8329 | 0.8546 | 0.8546 | 0.8546 |
| Insula_L | 23.2809±2.7784 | 22.3657±3.5870 | 22.7009±3.1188 | 0.8857 | 0.6792 | 0.6792 | 0.6792 |
| Insula_R | 21.1490±3.5490 | 21.1166±4.1145 | 22.1470±3.3300 | 0.7857 | 0.9897 | 0.4272 | 0.4272 |
| Cingulum_Ant_L | 18.2638±2.9719 | 18.8308±2.7841 | 18.1276±2.2720 | 0.7857 | 0.8369 | 0.8369 | 0.8369 |
| Cingulum_Ant_R | 16.7776±3.2293 | 16.8681±3.3660 | 16.6463±3.0500 | 0.9621 | 0.9968 | 0.9968 | 0.9968 |
| Cingulum_Mid_L | 12.3672±2.2796 | 13.8865±2.8335 | 13.5385±2.1898 | 0.7231 | 0.6747 | 0.5148 | 0.6747 |
| Cingulum_Mid_R | 14.0406±1.6118 | 15.5177±2.8752 | 14.5123±2.6200 | 0.7231 | 0.6649 | 0.6649 | 0.6099 |
| Cingulum_Post_L | 17.4950±3.9111 | 20.2697±3.6201 | 18.9968±2.7410 | 0.4140 | 0.1017 | 0.1017 | 0.2220 |
| Cingulum_Post_R | 9.6919±3.2433 | 9.4630±3.8964 | 9.0434±3.1252 | 0.9624 | 0.9889 | 0.9889 | 0.9889 |
| Hippocampus_L | 10.2479±3.5626 | 14.2541±5.3304 | 14.2487±4.8944 | 0.5515 | 0.0477 | 0.1275 | 0.9386 |
| Hippocampus_R | 23.0891±3.7264 | 25.8096±4.2394 | 24.8746±4.6327 | 0.5515 | 0.0999 | 0.3468 | 0.5686 |
| ParaHippocampal_L | 21.7535±3.0823 | 22.9616±3.2845 | 21.7287±3.2467 | 0.7231 | 0.9319 | 0.9413 | 0.3156 |
| ParaHippocampal_R | 13.4142±3.6097 | 13.9934±3.1568 | 13.1553±3.1139 | 0.7857 | 0.7849 | 0.7849 | 0.6966 |
| Amygdala_L | 9.5932±3.0752 | 10.2412±3.2095 | 12.5352±4.6955 | 0.3369 | 0.4705 | 0.0674 | 0.0674 |
| Amygdala_R | 6.9292±3.8812 | 8.8726±5.0007 | 9.7866±3.9490 | 0.7231 | 0.4075 | 0.4075 | 0.4075 |
| Calcarine_L | 30.5711±4.6631 | 30.1122±4.2377 | 28.0810±4.2687 | 0.7231 | 0.8716 | 0.4428 | 0.3042 |
| Calcarine_R | 29.4648±3.3668 | 29.5631±3.8385 | 28.3964±3.0350 | 0.7491 | 0.5674 | 0.4607 | 0.4607 |
| Cuneus_L | 20.9152±3.3366 | 22.9436±4.1988 | 22.5781±3.7604 | 0.7231 | 0.3959 | 0.3657 | 0.8195 |
| Cuneus_R | 16.7898±2.7403 | 15.8919±3.8391 | 16.5281±3.7175 | 0.8658 | 0.7545 | 0.7545 | 0.7545 |
| Lingual_L | 15.2344±3.5649 | 15.2094±3.3662 | 13.4384±2.9881 | 0.4390 | 0.6681 | 0.1544 | 0.0897 |
| Lingual_R | 23.0447±3.9465 | 24.3584±3.5415 | 21.7595±3.3085 | 0.3369 | 0.3943 | 0.0396 | 0.0321 |
| Occipital_Sup_L | 18.8495±3.9582 | 18.8403±3.8310 | 18.9651±5.0835 | 0.8856 | 0.7409 | 0.7409 | 0.7409 |
| Occipital_Sup_R | 17.3997±4.6695 | 15.2554±3.2796 | 15.5168±2.5394 | 0.6237 | 0.1961 | 0.1623 | 0.6945 |
| Occipital_Mid_L | 15.0944±5.0481 | 12.0167±3.5317 | 10.6648±3.4603 | 0.1800 | 0.0186 | 0.0186 | 0.2142 |
| Occipital_Mid_R | 16.5436±4.1116 | 15.7991±2.4550 | 16.5993±3.4996 | 0.7231 | 0.3775 | 0.3775 | 0.3775 |
| Occipital_Inf_L | 4.1026±2.2062 | 3.8609±2.3580 | 3.5336±2.1580 | 0.8857 | 0.5868 | 0.5868 | 0.5868 |
| Occipital_Inf_R | 14.4314±3.7947 | 14.0299±3.0192 | 13.7732±2.1951 | 0.6885 | 0.4271 | 0.4271 | 0.6191 |
| Fusiform_L | 23.4950±2.8183 | 23.4710±2.6118 | 22.5561±2.9743 | 0.7857 | 0.6133 | 0.6133 | 0.6133 |
| Fusiform_R | 17.8439±4.2314 | 18.3589±3.2751 | 18.1452±2.9573 | 0.9692 | 0.8013 | 0.8013 | 0.8013 |
| Postcentral_L | 22.1193±3.3731 | 20.0210±3.3376 | 20.8305±3.3016 | 0.7231 | 0.3876 | 0.3996 | 0.3996 |
| Postcentral_R | 25.5611±3.8170 | 22.6614±3.8957 | 23.0875±3.1762 | 0.7231 | 0.2034 | 0.3675 | 0.3675 |
| Parietal_Sup_L | 33.4082±4.1035 | 31.5646±3.8824 | 31.4124±4.1111 | 0.7231 | 0.1290 | 0.4672 | 0.9398 |
| Parietal_Sup_R | 29.4057±5.8202 | 25.4272±4.6699 | 26.9813±5.9554 | 0.6224 | 0.1272 | 0.1272 | 0.3109 |
| Parietal_Inf_L | 16.3353±1.9051 | 14.2816±1.9103 | 14.3686±2.0971 | 0.5515 | 0.0285 | 0.1028 | 0.5517 |
| Parietal_Inf_R | 9.1703±1.7235 | 8.0922±1.8555 | 8.0501±2.1473 | 0.8658 | 0.6021 | 0.6021 | 0.8308 |
| SupraMarginal_L | 13.8199±2.1062 | 13.6348±1.8961 | 13.3082±1.3349 | 0.8436 | 0.6817 | 0.6817 | 0.6817 |
| SupraMarginal_R | 14.4884±2.0861 | 13.3044±2.8146 | 12.4307±1.9272 | 0.6785 | 0.2845 | 0.2845 | 0.2845 |
| Angular_L | 15.1877±2.6577 | 16.0904±1.5898 | 15.5390±1.5485 | 0.7231 | 0.4560 | 0.9536 | 0.4560 |
| Angular_R | 13.4875±2.4651 | 13.0956±2.3652 | 13.9417±2.3188 | 0.7231 | 0.9591 | 0.6223 | 0.3513 |
| Precuneus_L | 45.5799±5.3444 | 46.1293±5.6120 | 45.7574±4.1414 | 0.9621 | 0.8953 | 0.8953 | 0.8953 |
| Precuneus_R | 43.6314±5.1538 | 43.5068±3.9810 | 43.4161±4.4688 | 0.9999 | 0.8975 | 0.8975 | 0.8975 |
| Paracentral_Lobule_L | 10.8286±2.4913 | 9.8708±2.1874 | 10.3267±2.0522 | 0.7231 | 0.0600 | 0.6634 | 0.5496 |
| Paracentral_Lobule_R | 15.9014±2.9320 | 14.7670±1.6144 | 15.7920±2.1105 | 0.5515 | 0.0273 | 0.5353 | 0.0897 |
| Caudate_L | 32.0002±3.4342 | 30.3711±4.7149 | 29.8562±3.2583 | 0.7857 | 0.6270 | 0.3429 | 0.7936 |
| Caudate_R | 22.3734±3.7061 | 19.2827±4.3703 | 19.8205±5.0305 | 0.6785 | 0.0603 | 0.7439 | 0.7439 |
| Putamen_L | 9.5816±4.2728 | 12.7551±5.3458 | 13.3393±4.9341 | 0.7231 | 0.6867 | 0.5379 | 0.6870 |
| Putamen_R | 26.0305±4.7447 | 25.3145±4.2799 | 25.8208±4.7278 | 0.8857 | 0.8151 | 0.8842 | 0.8842 |
| Pallidum_L | 8.3413±2.4165 | 7.8396±3.4097 | 9.9018±3.1422 | 0.2250 | 0.7349 | 0.0295 | 0.0210 |
| Pallidum_R | 11.2525±3.1205 | 11.5107±5.2121 | 12.7566±4.4377 | 0.7339 | 0.2819 | 0.0618 | 0.3350 |
| Thalamus_L | 20.1192±3.1485 | 17.3325±4.5345 | 17.6494±3.5816 | 0.7231 | 0.2562 | 0.2562 | 0.5872 |
| Thalamus_R | 14.3588±4.2418 | 12.1314±4.7871 | 12.4122±3.8044 | 0.7231 | 0.2008 | 0.2008 | 0.8737 |
| Heschl_L | 11.7898±1.5059 | 11.1347±1.0114 | 11.7326±1.6788 | 0.7231 | 0.1259 | 0.5818 | 0.1259 |
| Heschl_R | 1.6959±1.8247 | 0.9903±1.3175 | 0.8221±1.3880 | 0.7857 | 0.5549 | 0.5549 | 0.6138 |
| Temporal_Sup_L | 20.3520±3.7323 | 20.8552±5.1066 | 20.4331±2.9890 | 0.8857 | 0.8376 | 0.8376 | 0.8376 |
| Temporal_Sup_R | 18.3043±3.5658 | 18.9261±3.4836 | 18.0307±3.5370 | 0.8329 | 0.8534 | 0.8534 | 0.8534 |
| Temporal_Pole_Sup_L | 19.6786±3.6181 | 20.7442±4.1826 | 20.6724±3.1453 | 0.7231 | 0.4431 | 0.1704 | 0.9571 |
| Temporal_Pole_Sup_R | 21.5066±5.3811 | 22.3141±3.7627 | 20.7224±4.1611 | 0.7231 | 0.4831 | 0.6211 | 0.4665 |
| Temporal_Mid_L | 27.6473±3.5515 | 28.9153±4.3164 | 29.4365±3.1497 | 0.7724 | 0.6227 | 0.6227 | 0.7494 |
| Temporal_Mid_R | 23.2552±3.4046 | 23.6274±2.9463 | 23.7427±2.3995 | 0.7857 | 0.7761 | 0.1293 | 0.7761 |
| Temporal_Pole_Mid_L | 11.4239±3.3775 | 10.4323±3.6111 | 11.6780±3.5235 | 0.7231 | 0.5172 | 0.7580 | 0.5172 |
| Temporal_Pole_Mid_R | 8.7410±3.1845 | 6.6613±3.6800 | 6.5622±3.5773 | 0.7231 | 0.2803 | 0.2803 | 0.8561 |
| Temporal_Inf_L | 26.4725±4.7903 | 28.2345±4.2239 | 27.3732±3.6040 | 0.5515 | 0.1469 | 0.1469 | 0.6128 |
| Temporal_Inf_R | 22.4409±4.8023 | 22.0837±3.8317 | 22.7935±3.9056 | 0.7231 | 0.6176 | 0.5448 | 0.5448 |

^a^mean±standard deviation, controlling for age, gender, and education level.

^b^P-values from the permutation-based ANCOVA, controlling for age, gender, and education level between the three groups; the collected P-values were FDR-adjusted over 90 nodes.

^c^P-values from post-hoc tests using a three pairwise permutation-based ANCOVA, controlling for age, gender, and education level; the collected P-values were FDR-adjusted across the three pairwise comparisons.

**Supplementary Table S4. Group difference of the nodal strength**

| Nodes | HOA | LLD-MCI-A(+) | LLD-MCI-A(-) | Three-group  comparison | HOA  vs.  LLD-MCI-A(+) | HOA  vs.  LLD-MCI-A(-) | LLD-MCI-A(+)  vs.  LLD-MCI-A(-) |
| --- | --- | --- | --- | --- | --- | --- | --- |
| Precentral_L | 1560.1836±228.7449^a^ | 1334.4943±218.1709 | 1429.4172±205.4494 | 0.4326^b^ | 0.0303^c^ | 0.8155^c^ | 0.0377^c^ |
| Precentral_R | 1699.4039±201.8359 | 1580.8429±223.5440 | 1679.3618±251.9066 | 0.5585 | 0.2512 | 0.2512 | 0.1542 |
| Frontal_Sup_L | 1862.2126±276.2745 | 1712.8930±261.5260 | 1638.9757±210.0982 | 0.6145 | 0.2373 | 0.2925 | 0.3775 |
| Frontal_Sup_R | 2138.8250±371.8124 | 1832.0097±289.6579 | 1876.4135±239.5293 | 0.5859 | 0.2163 | 0.5583 | 0.5337 |
| Frontal_Sup_Orb_L | 586.9828±97.2649 | 540.1649±92.2959 | 574.7859±72.0827 | 0.5942 | 0.6948 | 0.6948 | 0.4041 |
| Frontal_Sup_Orb_R | 437.9955±128.4402 | 394.3196±94.3355 | 374.7332±81.8554 | 0.9204 | 0.9521 | 0.9521 | 0.9521 |
| Frontal_Mid_L | 1777.8861±253.9547 | 1607.9755±188.3112 | 1610.5406±205.0609 | 0.5585 | 0.0774 | 0.2112 | 0.9161 |
| Frontal_Mid_R | 1583.1044±266.1529 | 1312.4987±271.7920 | 1368.5702±203.9385 | 0.4834 | 0.2271 | 0.5707 | 0.4421 |
| Frontal_Mid_Orb_L | 425.4746±80.1488 | 406.8990±70.1855 | 395.8780±55.0360 | 0.9204 | 0.9563 | 0.9471 | 0.9563 |
| Frontal_Mid_Orb_R | 507.8136±60.2620 | 505.7929±87.5197 | 460.6368±69.4135 | 0.4834 | 0.3809 | 0.3825 | 0.2193 |
| Frontal_Inf_Oper_L | 789.3733±168.0212 | 773.8678±105.4911 | 772.3347±95.4240 | 0.9867 | 0.9559 | 0.9559 | 0.9559 |
| Frontal_Inf_Oper_R | 781.1128±121.7551 | 684.2597±114.6413 | 707.2847±117.0383 | 0.5950 | 0.5141 | 0.5141 | 0.5141 |
| Frontal_Inf_Tri_L | 1509.6030±252.0333 | 1390.7307±161.6126 | 1330.9205±131.7301 | 0.5690 | 0.6282 | 0.6138 | 0.5826 |
| Frontal_Inf_Tri_R | 924.3870±246.2365 | 719.6488±154.4316 | 707.6994±142.7575 | 0.3354 | 0.1123 | 0.1123 | 0.8636 |
| Frontal_Inf_Orb_L | 935.4897±157.9218 | 826.1450±126.2510 | 792.6390±140.5309 | 0.5690 | 0.3019 | 0.3019 | 0.4965 |
| Frontal_Inf_Orb_R | 868.0110±125.6880 | 691.9264±119.8962 | 640.1641±102.1215 | 0.0360 | 0.0244 | 0.0012 | 0.1606 |
| Rolandic_Oper_L | 772.2151±153.8603 | 720.2173±90.8684 | 713.9095±98.9340 | 0.7867 | 0.7824 | 0.7824 | 0.9191 |
| Rolandic_Oper_R | 549.5594±128.9357 | 528.5210±165.3467 | 528.5865±157.6040 | 0.9204 | 0.9220 | 0.9220 | 0.9220 |
| Supp_Motor_Area_L | 2318.1593±347.0505 | 2282.8373±270.0138 | 2252.9913±218.8025 | 0.9151 | 0.8637 | 0.7752 | 0.8637 |
| Supp_Motor_Area_R | 2665.0387±373.4148 | 2706.5191±269.9480 | 2598.0750±198.1036 | 0.6736 | 0.8208 | 0.9504 | 0.3480 |
| Olfactory_L | 235.0889±68.3192 | 284.9668±65.6558 | 277.3639±56.9409 | 0.5585 | 0.2891 | 0.2891 | 0.6070 |
| Olfactory_R | 83.8878±77.5247 | 101.0660±56.0202 | 75.3443±61.6324 | 0.6592 | 0.7350 | 0.8224 | 0.2850 |
| Frontal_Sup_Medial_L | 1583.6274±262.3686 | 1403.1211±250.1593 | 1333.5289±161.1211 | 0.6592 | 0.3763 | 0.3763 | 0.3763 |
| Frontal_Sup_Medial_R | 1535.8861±241.3875 | 1520.0537±259.4110 | 1444.3258±132.0759 | 0.6592 | 0.4389 | 0.6866 | 0.4595 |
| Frontal_Med_Orb_L | 503.3869±125.7174 | 550.3310±102.1293 | 509.1026±100.2100 | 0.4453 | 0.0921 | 0.4551 | 0.3455 |
| Frontal_Med_Orb_R | 589.9274±142.6102 | 592.0423±90.4084 | 553.4185±118.8498 | 0.7774 | 0.6509 | 0.6509 | 0.6180 |
| Rectus_L | 644.9788±85.5234 | 612.7180±107.5047 | 618.9568±103.2548 | 0.8211 | 0.7811 | 0.7811 | 0.9202 |
| Rectus_R | 681.9836±90.2716 | 691.0269±125.9208 | 681.7994±116.7819 | 0.9626 | 0.9707 | 0.9707 | 0.9707 |
| Insula_L | 1096.6789±181.6379 | 1038.6445±153.8217 | 985.3911±135.3505 | 0.6592 | 0.9050 | 0.5637 | 0.5637 |
| Insula_R | 788.2960±159.3852 | 641.6447±146.4464 | 695.4844±137.8709 | 0.4834 | 0.4500 | 0.5920 | 0.2265 |
| Cingulum_Ant_L | 586.1707±136.5943 | 556.1949±132.4173 | 523.8792±90.9513 | 0.7867 | 0.5411 | 0.5411 | 0.5411 |
| Cingulum_Ant_R | 595.7002±143.4121 | 531.0943±148.8819 | 463.9375±93.4451 | 0.2250 | 0.1041 | 0.0213 | 0.1041 |
| Cingulum_Mid_L | 577.2280±144.2904 | 467.2880±189.4557 | 465.3205±153.5460 | 0.6734 | 0.1329 | 0.9254 | 0.9254 |
| Cingulum_Mid_R | 925.1834±176.9333 | 791.4180±196.6316 | 717.9654±141.9992 | 0.1650 | 0.0306 | 0.0603 | 0.1239 |
| Cingulum_Post_L | 496.7308±88.5224 | 482.0947±71.5408 | 456.5524±38.9458 | 0.7119 | 0.9946 | 0.9946 | 0.5283 |
| Cingulum_Post_R | 220.8554±67.1853 | 206.3579±78.6128 | 187.8927±68.1939 | 0.8490 | 0.8574 | 0.8574 | 0.8574 |
| Hippocampus_L | 809.9551±115.1108 | 848.2206±89.0422 | 852.4271±101.0262 | 0.5942 | 0.2727 | 0.3978 | 0.8772 |
| Hippocampus_R | 868.8496±165.9259 | 921.3094±142.1974 | 919.1906±108.8353 | 0.5859 | 0.1462 | 0.1462 | 0.9870 |
| ParaHippocampal_L | 889.2589±125.1537 | 872.6004±152.9064 | 899.5832±71.0393 | 0.7867 | 0.8912 | 0.6787 | 0.6787 |
| ParaHippocampal_R | 668.4754±95.3998 | 602.8809±154.5607 | 645.2020±111.0247 | 0.6592 | 0.3443 | 0.6523 | 0.3443 |
| Amygdala_L | 143.0276±39.4047 | 148.0431±40.4507 | 169.3750±49.3132 | 0.2767 | 0.3896 | 0.1512 | 0.1512 |
| Amygdala_R | 71.8811±27.3070 | 88.1051±54.3743 | 104.3045±39.5923 | 0.4834 | 0.3037 | 0.3037 | 0.3037 |
| Calcarine_L | 2579.7820±196.4793 | 2731.2453±288.6206 | 2442.0984±258.1014 | 0.0090 | 0.0324 | 0.6690 | 0.0021 |
| Calcarine_R | 1713.1920±117.5937 | 1753.0136±197.6841 | 1615.2596±177.8757 | 0.2448 | 0.8323 | 0.8323 | 0.0636 |
| Cuneus_L | 1377.5752±178.8585 | 1533.8225±272.7556 | 1377.9669±206.0567 | 0.2250 | 0.0648 | 0.5593 | 0.0648 |
| Cuneus_R | 1358.2198±179.9159 | 1343.2960±211.4412 | 1273.8796±170.9296 | 0.7127 | 0.9494 | 0.9494 | 0.8949 |
| Lingual_L | 1410.2310±172.7298 | 1469.9840±212.5978 | 1324.1760±181.4386 | 0.2757 | 0.6967 | 0.9072 | 0.0588 |
| Lingual_R | 1502.3717±170.4121 | 1552.3932±177.9991 | 1406.2390±107.3884 | 0.1044 | 0.5342 | 0.5819 | 0.0051 |
| Occipital_Sup_L | 1105.8743±182.2863 | 1100.5962±200.1935 | 1112.3291±151.9843 | 0.7867 | 0.9480 | 0.5781 | 0.9261 |
| Occipital_Sup_R | 879.8545±102.4371 | 862.4700±148.2769 | 904.9298±102.5837 | 0.6779 | 0.8596 | 0.3695 | 0.3695 |
| Occipital_Mid_L | 1589.4843±194.9897 | 1583.7486±184.2492 | 1555.3203±192.1806 | 0.9204 | 0.9072 | 0.9072 | 0.9072 |
| Occipital_Mid_R | 505.2995±129.1864 | 455.1977±158.4043 | 453.5452±111.0093 | 0.7127 | 0.8442 | 0.8528 | 0.9703 |
| Occipital_Inf_L | 318.9806±101.5687 | 279.7365±112.9295 | 254.8088±88.2817 | 0.7867 | 0.6897 | 0.9830 | 0.7989 |
| Occipital_Inf_R | 419.4444±112.9084 | 317.1917±85.5157 | 301.9047±93.0418 | 0.0960 | 0.0211 | 0.0211 | 0.6086 |
| Fusiform_L | 1813.5925±236.3242 | 1725.0458±207.9197 | 1637.9677±163.6754 | 0.6592 | 0.8618 | 0.8618 | 0.5211 |
| Fusiform_R | 1409.2767±161.6609 | 1232.9143±200.7309 | 1223.8864±105.1840 | 0.3354 | 0.0558 | 0.1905 | 0.9582 |
| Postcentral_L | 1218.4864±193.1315 | 1176.3582±200.1708 | 1143.9345±196.1892 | 0.9204 | 0.8255 | 0.8255 | 0.8708 |
| Postcentral_R | 1592.7906±245.2361 | 1435.1275±161.0028 | 1491.3575±198.5099 | 0.6356 | 0.1477 | 0.5851 | 0.1477 |
| Parietal_Sup_L | 1013.5343±158.2222 | 928.6772±205.4903 | 978.0161±143.8230 | 0.6855 | 0.4357 | 0.9516 | 0.4357 |
| Parietal_Sup_R | 1385.6242±257.8042 | 1187.3587±163.6797 | 1261.4565±184.6310 | 0.4453 | 0.1527 | 0.3817 | 0.1544 |
| Parietal_Inf_L | 1006.5436±224.0924 | 871.3427±197.0415 | 901.3326±178.8694 | 0.7867 | 0.4761 | 0.4974 | 0.4974 |
| Parietal_Inf_R | 936.7828±147.5439 | 852.6055±140.7896 | 859.4417±120.2003 | 0.8920 | 0.6492 | 0.8834 | 0.8834 |
| SupraMarginal_L | 524.2209±93.6160 | 460.0092±100.2394 | 452.0681±103.2609 | 0.6356 | 0.2784 | 0.3742 | 0.9046 |
| SupraMarginal_R | 898.2496±172.7492 | 833.1361±150.9029 | 818.2548±132.3594 | 0.7119 | 0.4182 | 0.8871 | 0.8871 |
| Angular_L | 755.5179±125.4892 | 801.1168±115.1745 | 782.1268±143.3703 | 0.6855 | 0.7780 | 0.5967 | 0.7780 |
| Angular_R | 1060.4017±172.3447 | 1033.2366±175.1438 | 1094.9313±201.4004 | 0.5942 | 0.6311 | 0.4309 | 0.4309 |
| Precuneus_L | 1800.1015±227.9212 | 1634.5561±212.7459 | 1603.7728±229.7739 | 0.6048 | 0.1650 | 0.8236 | 0.8236 |
| Precuneus_R | 2543.3296±229.0870 | 2487.1493±221.9553 | 2416.3341±253.4008 | 0.7867 | 0.8441 | 0.8441 | 0.8441 |
| Paracentral_Lobule_L | 462.2138±175.1482 | 315.4844±168.2892 | 335.6590±127.7170 | 0.5585 | 0.0843 | 0.7805 | 0.6086 |
| Paracentral_Lobule_R | 443.2410±154.2272 | 334.8448±79.9077 | 340.5514±70.4796 | 0.1916 | 0.0135 | 0.0577 | 0.5133 |
| Caudate_L | 498.2838±90.3484 | 546.8803±151.6959 | 497.3969±88.8505 | 0.5585 | 0.4262 | 0.5928 | 0.4262 |
| Caudate_R | 424.9939±89.8776 | 498.1507±188.6061 | 482.9618±130.9920 | 0.6734 | 0.6693 | 0.0084 | 0.6693 |
| Putamen_L | 569.6577±149.6457 | 537.8017±162.7726 | 465.0397±113.0762 | 0.5859 | 0.7712 | 0.5804 | 0.2712 |
| Putamen_R | 627.7474±159.2153 | 593.9551±156.3181 | 608.3276±113.1652 | 0.9204 | 0.8579 | 0.8579 | 0.8579 |
| Pallidum_L | 320.5740±79.1384 | 292.6235±88.8872 | 301.6316±101.5060 | 0.9204 | 0.3033 | 0.6576 | 0.6576 |
| Pallidum_R | 396.9691±93.9409 | 394.2347±115.5189 | 409.7512±70.9548 | 0.7811 | 0.7108 | 0.1536 | 0.7108 |
| Thalamus_L | 47.4065±150.4699 | 24.4798±150.3474 | 76.1290±102.5635 | 0.3032 | 0.8779 | 0.2661 | 0.0231 |
| Thalamus_R | 284.4271±156.0482 | 315.8977±146.7058 | 227.9726±113.6637 | 0.4496 | 0.9844 | 0.9844 | 0.0375 |
| Heschl_L | 307.0923±50.4823 | 294.9731±34.2057 | 289.3815±40.1399 | 0.8490 | 0.6780 | 0.6780 | 0.6780 |
| Heschl_R | 142.4295±45.2975 | 141.9833±47.9529 | 163.9310±63.3413 | 0.5690 | 0.7536 | 0.4236 | 0.4176 |
| Temporal_Sup_L | 971.6308±164.3152 | 865.0488±134.9370 | 840.1240±113.7965 | 0.4453 | 0.1071 | 0.1772 | 0.5201 |
| Temporal_Sup_R | 1446.0816±203.0479 | 1255.4447±209.9656 | 1295.3830±200.0551 | 0.5585 | 0.0999 | 0.5192 | 0.3430 |
| Temporal_Pole_Sup_L | 606.2910±108.4095 | 568.5273±137.5542 | 532.7022±95.0029 | 0.7867 | 0.8925 | 0.8925 | 0.8925 |
| Temporal_Pole_Sup_R | 262.6253±86.3359 | 227.7344±101.2152 | 185.7803±71.1235 | 0.5585 | 0.8497 | 0.1527 | 0.1527 |
| Temporal_Mid_L | 2120.3282±259.5399 | 1910.5209±234.9930 | 1910.5213±184.8393 | 0.5585 | 0.1479 | 0.9305 | 0.9305 |
| Temporal_Mid_R | 1745.9577±251.3708 | 1397.9040±252.6211 | 1460.9755±216.7025 | 0.1044 | 0.0051 | 0.0264 | 0.1541 |
| Temporal_Pole_Mid_L | 427.9272±104.3549 | 379.5500±89.9360 | 416.4354±85.5097 | 0.5942 | 0.5007 | 0.9763 | 0.4056 |
| Temporal_Pole_Mid_R | 243.8419±82.0871 | 199.6206±104.4783 | 190.3658±84.1582 | 0.7119 | 0.3426 | 0.3324 | 0.7456 |
| Temporal_Inf_L | 1318.4879±174.7806 | 1220.8554±156.433 | 1200.5215±129.4977 | 0.9204 | 0.8577 | 0.8577 | 0.8577 |
| Temporal_Inf_R | 1069.2091±185.0968 | 909.9269±174.7059 | 955.0891±142.2215 | 0.5585 | 0.1842 | 0.9644 | 0.2241 |

^a^mean±standard deviation, controlling for age, gender, and education level.

^b^P-values from the permutation-based ANCOVA, controlling for age, gender, and education level between the three groups; the collected P-values were FDR-adjusted over 90 nodes.

^c^P-values from post-hoc tests using a three pairwise permutation-based ANCOVA, controlling for age, gender, and education level; the collected P-values were FDR-adjusted across the three pairwise comparisons.

**Supplementary Table S5. Group difference of the clustering coefficient**

| Nodes | HOA | LLD-MCI-A(+) | LLD-MCI-A(-) | Three-group  comparison | HOA  vs.  LLD-MCI-A(+) | HOA  vs.  LLD-MCI-A(-) | LLD-MCI-A(+)  vs.  LLD-MCI-A(-) |
| --- | --- | --- | --- | --- | --- | --- | --- |
| Precentral_L | 13.8351±4.5692^a^ | 9.8201±2.8075 | 10.3161±2.9137 | 0.4415^b^ | 0.1047^c^ | 0.3627^c^ | 0.5325^c^ |
| Precentral_R | 27.6040±4.9280 | 27.2861±6.3799 | 25.9093±4.0991 | 0.8640 | 0.9550 | 0.9550 | 0.9550 |
| Frontal_Sup_L | 21.3508±3.4681 | 18.4375±2.9607 | 18.1775±3.0599 | 0.4508 | 0.1224 | 0.1224 | 0.9082 |
| Frontal_Sup_R | 15.0741±2.0387 | 13.5832±2.3488 | 13.0409±2.0830 | 0.6266 | 0.6036 | 0.6456 | 0.6315 |
| Frontal_Sup_Orb_L | 10.8332±1.6999 | 11.1719±1.4468 | 11.0423±1.3692 | 0.8275 | 0.7659 | 0.7659 | 0.7659 |
| Frontal_Sup_Orb_R | 9.3677±1.6894 | 8.8739±1.1313 | 9.2105±1.2650 | 0.7247 | 0.7970 | 0.2937 | 0.3503 |
| Frontal_Mid_L | 41.4994±13.8245 | 35.0399±12.0194 | 35.8224±10.5603 | 0.7247 | 0.3075 | 0.8019 | 0.8019 |
| Frontal_Mid_R | 20.3370±8.6833 | 17.8208±8.8472 | 20.6149±11.7155 | 0.7247 | 0.8335 | 0.8335 | 0.8335 |
| Frontal_Mid_Orb_L | 13.9049±6.0543 | 13.6217±5.3148 | 12.5044±2.7573 | 0.8640 | 0.9441 | 0.9441 | 0.9441 |
| Frontal_Mid_Orb_R | 16.4876±6.8632 | 16.0765±3.9092 | 18.4753±5.1973 | 0.5391 | 0.9244 | 0.2102 | 0.2102 |
| Frontal_Inf_Oper_L | 33.4507±6.0255 | 35.2329±7.5432 | 31.8284±4.2907 | 0.4514 | 0.7335 | 0.5673 | 0.1488 |
| Frontal_Inf_Oper_R | 53.5878±9.205 | 54.7361±7.9386 | 54.4539±8.3586 | 0.9885 | 0.9610 | 0.9610 | 0.9610 |
| Frontal_Inf_Tri_L | 6.2950±8.0971 | 9.2053±7.1634 | 6.7823±8.9038 | 0.7247 | 0.6473 | 0.6473 | 0.6473 |
| Frontal_Inf_Tri_R | 20.1593±9.9215 | 20.4769±8.5255 | 19.5470±7.6598 | 0.9032 | 0.6917 | 0.6917 | 0.6917 |
| Frontal_Inf_Orb_L | 0.8203±3.5352 | 0.1119±3.1482 | 0.1616±2.8932 | 0.9885 | 0.9374 | 0.9374 | 0.9374 |
| Frontal_Inf_Orb_R | 9.9902±2.2164 | 10.5682±2.2003 | 9.7157±2.1290 | 0.6699 | 0.6063 | 0.6063 | 0.5145 |
| Rolandic_Oper_L | 22.9352±5.9579 | 19.9729±4.0122 | 19.2479±3.9310 | 0.5431 | 0.2508 | 0.2508 | 0.4843 |
| Rolandic_Oper_R | 27.8035±3.9408 | 28.9526±4.9843 | 28.9791±4.4464 | 0.7247 | 0.6847 | 0.6847 | 0.7927 |
| Supp_Motor_Area_L | 21.7047±3.2457 | 18.9882±3.6613 | 18.0534±3.3096 | 0.4415 | 0.1620 | 0.0687 | 0.5969 |
| Supp_Motor_Area_R | 19.3888±4.3991 | 18.0489±3.378 | 18.0057±4.2390 | 0.8640 | 0.8956 | 0.8956 | 0.8956 |
| Olfactory_L | 6.5016±1.4769 | 7.6457±1.5405 | 7.1956±1.6077 | 0.4415 | 0.0564 | 0.4274 | 0.4274 |
| Olfactory_R | 6.5321±1.1580 | 7.8379±1.4390 | 7.7648±1.5669 | 0.4508 | 0.0603 | 0.4105 | 0.7063 |
| Frontal_Sup_Medial_L | 15.4465±2.8438 | 15.4198±3.0467 | 13.4655±1.8693 | 0.4415 | 0.6628 | 0.2607 | 0.0327 |
| Frontal_Sup_Medial_R | 12.9358±3.5645 | 10.9383±2.7301 | 10.0644±2.7272 | 0.7247 | 0.5915 | 0.5915 | 0.5915 |
| Frontal_Med_Orb_L | 22.4189±4.2305 | 22.7606±2.8533 | 21.3484±3.3018 | 0.7247 | 0.4855 | 0.8361 | 0.2649 |
| Frontal_Med_Orb_R | 15.3016±1.9895 | 15.5873±3.4965 | 15.2021±3.7727 | 0.9226 | 0.6998 | 0.6998 | 0.7016 |
| Rectus_L | 10.6832±2.5032 | 12.2818±3.2999 | 11.5174±2.5541 | 0.5391 | 0.2757 | 0.4194 | 0.4194 |
| Rectus_R | 11.1154±1.6149 | 12.0471±2.7160 | 12.1848±3.0940 | 0.8906 | 0.9745 | 0.9745 | 0.9745 |
| Insula_L | 19.3277±2.9911 | 20.3688±4.1203 | 18.8019±2.3521 | 0.5391 | 0.2461 | 0.9975 | 0.2461 |
| Insula_R | 16.0428±4.9381 | 15.2466±5.4701 | 14.0886±2.2734 | 0.8640 | 0.6976 | 0.6976 | 0.6976 |
| Cingulum_Ant_L | 14.3145±2.6984 | 13.8836±2.9662 | 13.1139±1.9308 | 0.7247 | 0.5405 | 0.5405 | 0.5405 |
| Cingulum_Ant_R | 16.5023±2.7972 | 15.6429±3.0793 | 14.1855±1.8745 | 0.4508 | 0.4481 | 0.3237 | 0.1983 |
| Cingulum_Mid_L | 19.3521±4.5161 | 14.7703±5.6197 | 14.7148±4.2426 | 0.4415 | 0.0795 | 0.3240 | 0.9037 |
| Cingulum_Mid_R | 23.6508±4.6159 | 20.0539±3.5005 | 20.4957±3.5935 | 0.4415 | 0.1278 | 0.4536 | 0.6884 |
| Cingulum_Post_L | 13.1984±3.7427 | 11.0792±3.5838 | 10.6183±3.2842 | 0.6065 | 0.3561 | 0.3749 | 0.6420 |
| Cingulum_Post_R | 14.0415±3.0265 | 14.0999±3.1274 | 13.0600±2.2497 | 0.7247 | 0.9964 | 0.5660 | 0.5660 |
| Hippocampus_L | 19.8824±3.7956 | 17.1710±2.8582 | 16.7556±2.4620 | 0.4415 | 0.1038 | 0.1038 | 0.6025 |
| Hippocampus_R | 10.6337±2.1085 | 10.1159±1.5520 | 9.8833±1.0150 | 0.7247 | 0.7751 | 0.7751 | 0.7751 |
| ParaHippocampal_L | 11.7532±3.1378 | 11.2417±4.9588 | 11.9039±4.0131 | 0.7800 | 0.8213 | 0.8572 | 0.8213 |
| ParaHippocampal_R | 16.6557±3.6353 | 15.4356±3.2919 | 15.6617±3.0365 | 0.8906 | 0.7312 | 0.7312 | 0.7312 |
| Amygdala_L | 23.3913±3.8900 | 23.8180±3.1119 | 22.0571±2.9661 | 0.5391 | 0.5075 | 0.2628 | 0.1440 |
| Amygdala_R | 8.0944±2.5619 | 7.6715±3.0803 | 6.7513±1.6134 | 0.7247 | 0.9178 | 0.9078 | 0.6045 |
| Calcarine_L | 20.6438±3.9034 | 22.3695±4.7075 | 20.6306±3.4342 | 0.6288 | 0.4132 | 0.4132 | 0.4132 |
| Calcarine_R | 14.4755±4.4014 | 15.3019±5.3892 | 13.6541±4.1330 | 0.7247 | 0.8425 | 0.4725 | 0.4725 |
| Cuneus_L | 27.9305±8.4191 | 27.1620±8.3819 | 23.5261±4.9310 | 0.4415 | 0.9188 | 0.1188 | 0.1188 |
| Cuneus_R | 35.0155±5.6647 | 37.6328±7.0447 | 33.8613±4.9042 | 0.4415 | 0.2282 | 0.5131 | 0.1563 |
| Lingual_L | 30.9109±5.0927 | 33.1926±4.9487 | 31.7815±3.3565 | 0.4415 | 0.1245 | 0.1280 | 0.3998 |
| Lingual_R | 15.4636±3.2835 | 15.3763±4.0348 | 15.4052±2.9302 | 0.8273 | 0.9615 | 0.0588 | 0.9615 |
| Occipital_Sup_L | 19.3046±5.5722 | 18.4278±6.0978 | 17.4943±5.0569 | 0.8129 | 0.7488 | 0.7488 | 0.7488 |
| Occipital_Sup_R | 18.2177±4.1712 | 20.1869±4.0792 | 19.6725±3.4636 | 0.7247 | 0.4023 | 0.1428 | 0.5735 |
| Occipital_Mid_L | 41.2267±7.2539 | 45.5373±7.3684 | 45.1961±4.7564 | 0.3735 | 0.0304 | 0.0126 | 0.9488 |
| Occipital_Mid_R | 14.0984±6.5271 | 13.6893±6.9444 | 12.2473±5.4361 | 0.8640 | 0.8571 | 0.7181 | 0.7181 |
| Occipital_Inf_L | 70.4294±17.6229 | 70.6925±16.1998 | 66.7205±12.4386 | 0.8640 | 0.8933 | 0.8933 | 0.8933 |
| Occipital_Inf_R | 3.3145±10.9852 | 4.2721±11.8629 | 6.8041±7.5054 | 0.7247 | 0.4877 | 0.4877 | 0.4999 |
| Fusiform_L | 23.5477±6.1375 | 23.7958±5.3899 | 22.4718±5.8999 | 0.8640 | 0.5211 | 0.7192 | 0.7192 |
| Fusiform_R | 19.7355±4.5839 | 18.0488±3.5078 | 17.6880±3.4440 | 0.8640 | 0.9345 | 0.9345 | 0.9345 |
| Postcentral_L | 13.7842±2.1019 | 14.0102±2.4720 | 13.4056±2.8504 | 0.8640 | 0.9520 | 0.9520 | 0.9520 |
| Postcentral_R | 16.9502±4.2703 | 18.0981±5.6357 | 16.8447±3.8045 | 0.8010 | 0.6702 | 0.6702 | 0.6702 |
| Parietal_Sup_L | 2.2174±3.6240 | 0.5667±8.2539 | 0.7820±9.1681 | 0.6309 | 0.2736 | 0.2736 | 0.5019 |
| Parietal_Sup_R | 15.0466±3.7680 | 16.7295±5.5534 | 17.0601±5.8459 | 0.6288 | 0.3132 | 0.3132 | 0.6681 |
| Parietal_Inf_L | 23.6105±6.3902 | 27.3715±7.5910 | 29.0488±7.1781 | 0.4415 | 0.1548 | 0.0105 | 0.4964 |
| Parietal_Inf_R | 69.6971±12.5387 | 71.1159±13.1891 | 74.3836±14.2018 | 0.8010 | 0.6801 | 0.6801 | 0.6801 |
| SupraMarginal_L | 32.1959±8.3642 | 28.3675±7.1543 | 29.4548±6.9839 | 0.8275 | 0.6783 | 0.9162 | 0.6783 |
| SupraMarginal_R | 41.7159±5.8962 | 43.0222±6.3295 | 46.2056±9.1046 | 0.5391 | 0.8225 | 0.2669 | 0.2669 |
| Angular_L | 23.1349±8.6699 | 22.6582±7.5533 | 25.9247±12.8345 | 0.7397 | 0.3202 | 0.3202 | 0.3202 |
| Angular_R | 42.7633±9.1599 | 41.3845±7.4820 | 41.4967±5.8583 | 0.8640 | 0.9868 | 0.9868 | 0.9868 |
| Precuneus_L | 7.7385±1.8142 | 7.4165±2.2515 | 6.5551±1.8974 | 0.6298 | 0.6195 | 0.6195 | 0.5058 |
| Precuneus_R | 9.0898±2.3092 | 8.7198±2.1160 | 7.8568±2.4962 | 0.7247 | 0.6998 | 0.6998 | 0.6998 |
| Paracentral_Lobule_L | 18.2917±4.9950 | 16.5065±5.0597 | 17.0244±4.5095 | 0.8640 | 0.8318 | 0.2097 | 0.7954 |
| Paracentral_Lobule_R | 13.7690±4.2621 | 12.3940±4.3219 | 10.6313±3.7658 | 0.6699 | 0.6958 | 0.8581 | 0.6462 |
| Caudate_L | 4.5882±1.0802 | 5.7790±1.9533 | 5.2128±1.0363 | 0.4415 | 0.1941 | 0.2126 | 0.2126 |
| Caudate_R | 6.3947±1.3306 | 7.3503±1.7874 | 7.0267±0.9450 | 0.4508 | 0.2013 | 0.1263 | 0.4414 |
| Putamen_L | 18.8889±3.4817 | 17.0643±2.1388 | 15.5786±1.7524 | 0.4415 | 0.4705 | 0.2610 | 0.0501 |
| Putamen_R | 11.8083±2.7174 | 11.4165±2.4459 | 10.7765±1.5217 | 0.8610 | 0.7603 | 0.7603 | 0.7603 |
| Pallidum_L | 27.1902±2.9873 | 28.4575±3.7485 | 24.1069±2.7305 | 0.0090 | 0.7259 | 0.0711 | 0.0003 |
| Pallidum_R | 16.0838±3.8989 | 16.1697±5.1603 | 14.5021±2.8996 | 0.7247 | 0.2819 | 0.2819 | 0.2819 |
| Thalamus_L | 2.4430±1.9347 | 3.3135±3.1425 | 1.2743±1.9099 | 0.3735 | 0.4031 | 0.9815 | 0.0198 |
| Thalamus_R | 1.9544±2.0219 | 2.9358±3.3008 | 0.8396±1.6143 | 0.3735 | 0.3166 | 0.8158 | 0.0114 |
| Heschl_L | 22.6435±10.0342 | 23.1271±6.8357 | 18.9577±6.4506 | 0.4514 | 0.7769 | 0.5649 | 0.0690 |
| Heschl_R | 67.5081±19.4410 | 70.2330±13.1523 | 73.7300±16.2395 | 0.7397 | 0.5129 | 0.5129 | 0.5129 |
| Temporal_Sup_L | 25.0868±9.6947 | 23.3855±6.5548 | 22.7739±8.2463 | 0.8640 | 0.7346 | 0.7346 | 0.7346 |
| Temporal_Sup_R | 22.4816±4.6093 | 20.2378±4.0677 | 22.2557±5.7024 | 0.6309 | 0.6044 | 0.6044 | 0.2199 |
| Temporal_Pole_Sup_L | 11.2258±2.1321 | 10.1104±1.9160 | 9.8914±2.4162 | 0.7247 | 0.3532 | 0.3532 | 0.7638 |
| Temporal_Pole_Sup_R | 5.5040±1.8192 | 4.7446±1.7958 | 5.3193±2.0318 | 0.7247 | 0.6060 | 0.8448 | 0.6060 |
| Temporal_Mid_L | 19.4007±2.7853 | 17.8730±4.9918 | 16.4285±3.2162 | 0.6309 | 0.4945 | 0.6521 | 0.4945 |
| Temporal_Mid_R | 19.7650±4.6106 | 16.4534±3.8128 | 16.8022±2.6913 | 0.4415 | 0.0735 | 0.0096 | 0.6005 |
| Temporal_Pole_Mid_L | 18.8353±4.0985 | 18.7724±3.3955 | 18.0033±2.3545 | 0.8640 | 0.9201 | 0.9201 | 0.9201 |
| Temporal_Pole_Mid_R | 13.1180±2.3338 | 13.2454±2.3771 | 13.1220±2.0305 | 0.9716 | 0.9663 | 0.9663 | 0.9663 |
| Temporal_Inf_L | 20.1266±7.2491 | 16.7054±3.7008 | 16.0782±3.8237 | 0.4415 | 0.4618 | 0.2376 | 0.5819 |
| Temporal_Inf_R | 9.1619±5.0458 | 8.0498±4.8375 | 6.9050±4.2139 | 0.5391 | 0.3147 | 0.3147 | 0.3147 |

^a^mean±standard deviation, controlling for age, gender, and education level.

^b^P-values from the permutation-based ANCOVA, controlling for age, gender, and education level between the three groups; the collected P-values were FDR-adjusted over 90 nodes.

^c^P-values from post-hoc tests using a three pairwise permutation-based ANCOVA, controlling for age, gender, and education level; the collected P-values were FDR-adjusted across the three pairwise comparisons.

**Supplementary Table S6. Group difference of the regional efficiency**

| Nodes | HOA | LLD-MCI-A(+) | LLD-MCI-A(-) | Three-group  comparison | HOA  vs.  LLD-MCI-A(+) | HOA  vs.  LLD-MCI-A(-) | LLD-MCI-A(+)  vs.  LLD-MCI-A(-) |
| --- | --- | --- | --- | --- | --- | --- | --- |
| Precentral_L | 70.2398±6.2701^a^ | 63.4032±6.7360 | 65.1160±6.1923 | 0.4819^b^ | 0.0273^c^ | 0.6459^c^ | 0.1646^c^ |
| Precentral_R | 70.0143±6.9922 | 63.1267±6.0320 | 65.8227±6.4872 | 0.4388 | 0.0474 | 0.6449 | 0.0474 |
| Frontal_Sup_L | 76.4233±6.5671 | 70.4159±5.9108 | 69.5724±6.4685 | 0.6056 | 0.0540 | 0.4677 | 0.8658 |
| Frontal_Sup_R | 74.9479±8.4621 | 66.3971±6.7263 | 67.9752±5.9975 | 0.4388 | 0.0909 | 0.8737 | 0.2756 |
| Frontal_Sup_Orb_L | 31.8457±4.3652 | 29.4303±3.5361 | 30.2798±2.7383 | 0.7205 | 0.4038 | 0.7173 | 0.4038 |
| Frontal_Sup_Orb_R | 31.2036±3.7428 | 28.9809±3.4760 | 27.9303±3.3707 | 0.7937 | 0.5275 | 0.5275 | 0.5275 |
| Frontal_Mid_L | 78.2499±6.4940 | 71.3298±5.2641 | 71.3482±6.3118 | 0.4388 | 0.0126 | 0.4069 | 0.6686 |
| Frontal_Mid_R | 75.9869±8.1048 | 67.4044±6.5664 | 69.4501±5.8953 | 0.4388 | 0.0903 | 0.9283 | 0.1233 |
| Frontal_Mid_Orb_L | 39.9255±4.0885 | 38.3983±4.1638 | 37.6365±2.9633 | 0.9422 | 0.8062 | 0.8062 | 0.8062 |
| Frontal_Mid_Orb_R | 48.4274±3.6699 | 47.6493±5.1511 | 46.2590±4.5248 | 0.7975 | 0.6706 | 0.6706 | 0.6706 |
| Frontal_Inf_Oper_L | 56.8062±6.1843 | 54.3754±4.0632 | 53.7640±4.6602 | 0.8115 | 0.5628 | 0.8295 | 0.8295 |
| Frontal_Inf_Oper_R | 57.5520±6.3592 | 51.3074±3.8315 | 51.9647±4.5889 | 0.4388 | 0.1785 | 0.4007 | 0.4007 |
| Frontal_Inf_Tri_L | 68.5014±6.5492 | 62.4464±4.6754 | 62.3076±5.1991 | 0.4388 | 0.1257 | 0.5701 | 0.8316 |
| Frontal_Inf_Tri_R | 59.6911±8.3139 | 53.2773±4.9633 | 54.6555±5.3804 | 0.5405 | 0.2689 | 0.8306 | 0.2689 |
| Frontal_Inf_Orb_L | 51.1062±5.0013 | 46.6753±4.7693 | 46.1658±3.7884 | 0.6603 | 0.1722 | 0.6999 | 0.9401 |
| Frontal_Inf_Orb_R | 49.5554±5.5784 | 43.9574±3.5355 | 43.2200±5.5492 | 0.4782 | 0.2943 | 0.3367 | 0.8472 |
| Rolandic_Oper_L | 45.6331±5.6236 | 41.5552±3.8234 | 40.3803±4.3706 | 0.4388 | 0.1362 | 0.3027 | 0.4313 |
| Rolandic_Oper_R | 44.5794±4.4416 | 39.7801±5.9110 | 40.3845±6.1591 | 0.7380 | 0.3195 | 0.6534 | 0.5337 |
| Supp_Motor_Area_L | 78.0344±8.3104 | 74.3289±7.0056 | 73.6296±6.3134 | 0.9663 | 0.9852 | 0.9852 | 0.9852 |
| Supp_Motor_Area_R | 79.5683±8.8990 | 75.9179±6.3506 | 74.1355±5.0873 | 0.7982 | 0.5818 | 0.6627 | 0.5818 |
| Olfactory_L | 22.6203±3.0118 | 23.9472±3.2252 | 22.5739±2.6082 | 0.4388 | 0.2438 | 0.6476 | 0.2438 |
| Olfactory_R | 17.3168±4.1755 | 18.0651±3.3002 | 16.1453±2.5388 | 0.4388 | 0.6645 | 0.9063 | 0.0912 |
| Frontal_Sup_Medial_L | 66.3403±7.3207 | 60.3793±6.4415 | 59.5673±5.6853 | 0.7937 | 0.3435 | 0.7296 | 0.9585 |
| Frontal_Sup_Medial_R | 62.7030±7.0381 | 59.4472±7.0157 | 57.7614±4.1381 | 0.8604 | 0.9116 | 0.8658 | 0.8658 |
| Frontal_Med_Orb_L | 30.4279±4.5540 | 29.1046±4.0013 | 27.8250±3.3156 | 0.8021 | 0.8567 | 0.8567 | 0.8567 |
| Frontal_Med_Orb_R | 28.5156±5.5374 | 26.5665±3.4083 | 25.3759±4.3013 | 0.7982 | 0.9369 | 0.7488 | 0.7488 |
| Rectus_L | 29.0576±3.2471 | 26.9500±2.7115 | 27.1143±2.3803 | 0.6880 | 0.4038 | 0.8112 | 0.8360 |
| Rectus_R | 30.6707±3.1190 | 28.7508±3.2675 | 28.9239±3.0179 | 0.7937 | 0.7227 | 0.9036 | 0.9036 |
| Insula_L | 52.9768±5.9198 | 48.1050±4.1725 | 46.8288±4.1170 | 0.4388 | 0.1394 | 0.1394 | 0.3549 |
| Insula_R | 42.7893±5.3280 | 36.3203±4.4085 | 37.3817±4.8965 | 0.4388 | 0.1077 | 0.2730 | 0.2653 |
| Cingulum_Ant_L | 40.1455±6.5537 | 34.9558±5.8143 | 34.7450±4.5752 | 0.6884 | 0.1668 | 0.7161 | 0.8764 |
| Cingulum_Ant_R | 42.6400±5.5658 | 37.9475±5.3417 | 35.3344±3.8670 | 0.4320 | 0.0256 | 0.0256 | 0.0755 |
| Cingulum_Mid_L | 46.8563±5.5506 | 39.7066±6.6223 | 39.7854±5.9402 | 0.4388 | 0.0069 | 0.4242 | 0.7234 |
| Cingulum_Mid_R | 60.3866±5.7791 | 55.6803±5.0816 | 52.8258±5.2226 | 0.4388 | 0.0300 | 0.1452 | 0.0996 |
| Cingulum_Post_L | 44.9837±6.0117 | 41.9320±4.6513 | 42.1682±3.0847 | 0.8710 | 0.7428 | 0.7428 | 0.7428 |
| Cingulum_Post_R | 34.4838±5.3748 | 32.6477±4.7566 | 33.5956±4.5763 | 0.6880 | 0.4316 | 0.2910 | 0.2910 |
| Hippocampus_L | 50.9255±5.1828 | 49.6156±4.2899 | 49.6433±3.2845 | 0.9496 | 0.8993 | 0.7602 | 0.8993 |
| Hippocampus_R | 50.5419±5.3582 | 49.3348±4.8186 | 49.8892±3.0318 | 0.7937 | 0.9411 | 0.1599 | 0.7149 |
| ParaHippocampal_L | 56.1960±3.5810 | 54.2782±4.8508 | 54.3121±3.4930 | 0.9619 | 0.6225 | 0.4662 | 0.7595 |
| ParaHippocampal_R | 52.8783±3.2961 | 49.9675±5.9759 | 50.4451±3.5074 | 0.8151 | 0.3659 | 0.3659 | 0.4781 |
| Amygdala_L | 24.1283±4.3720 | 23.9575±3.9793 | 24.6369±2.9424 | 0.6056 | 0.6033 | 0.2580 | 0.6033 |
| Amygdala_R | 15.7741±3.9638 | 15.1253±3.8954 | 17.2092±3.8523 | 0.4388 | 0.8908 | 0.2073 | 0.1566 |
| Calcarine_L | 79.7327±4.6107 | 78.4971±7.1728 | 75.1074±5.9111 | 0.5237 | 0.9759 | 0.9759 | 0.3798 |
| Calcarine_R | 81.0648±4.6685 | 79.9436±6.0407 | 77.3999±5.4002 | 0.6820 | 0.6526 | 0.6339 | 0.6006 |
| Cuneus_L | 69.0744±5.0477 | 68.8724±7.1001 | 66.2443±5.8539 | 0.6820 | 0.725 | 0.7250 | 0.7250 |
| Cuneus_R | 66.2976±4.4735 | 63.9254±6.7622 | 61.9716±5.0906 | 0.7937 | 0.7276 | 0.9539 | 0.7276 |
| Lingual_L | 69.2029±4.5018 | 67.1766±6.9471 | 65.0078±5.6446 | 0.7937 | 0.5921 | 0.5921 | 0.5921 |
| Lingual_R | 73.4533±4.8385 | 71.4257±6.0170 | 69.1115±4.6709 | 0.6880 | 0.6502 | 0.6502 | 0.6502 |
| Occipital_Sup_L | 63.4062±5.1609 | 63.2731±6.8516 | 62.2711±5.0893 | 0.7982 | 0.9950 | 0.5280 | 0.9950 |
| Occipital_Sup_R | 50.1218±4.7935 | 48.7764±5.9224 | 48.8584±3.8654 | 0.9663 | 0.8010 | 0.6684 | 0.8010 |
| Occipital_Mid_L | 63.9550±3.7894 | 62.6572±6.5359 | 61.5989±5.2248 | 0.9327 | 0.7702 | 0.7702 | 0.7702 |
| Occipital_Mid_R | 43.3574±4.2717 | 39.6527±6.2267 | 39.7309±3.9517 | 0.6069 | 0.3408 | 0.7651 | 0.8470 |
| Occipital_Inf_L | 41.9124±4.3148 | 39.1935±5.1046 | 38.4064±4.3100 | 0.8151 | 0.3024 | 0.9105 | 0.9105 |
| Occipital_Inf_R | 41.7308±3.7562 | 37.5154±4.2510 | 36.0374±4.0484 | 0.4320 | 0.0543 | 0.0516 | 0.2764 |
| Fusiform_L | 73.7006±4.7341 | 70.8313±5.7590 | 69.3733±4.9600 | 0.8151 | 0.6345 | 0.6345 | 0.6345 |
| Fusiform_R | 67.5688±4.6374 | 62.9762±6.1976 | 62.1571±4.5329 | 0.6846 | 0.1629 | 0.8794 | 0.8794 |
| Postcentral_L | 64.1560±5.5692 | 59.0431±6.4928 | 59.5175±5.9267 | 0.7982 | 0.0840 | 0.7771 | 0.5700 |
| Postcentral_R | 67.1271±6.5182 | 60.6872±5.3395 | 62.8121±6.3708 | 0.4388 | 0.0327 | 0.7429 | 0.0858 |
| Parietal_Sup_L | 58.9602±4.7748 | 54.1895±7.7781 | 55.0701±5.7131 | 0.7975 | 0.2646 | 0.8049 | 0.4725 |
| Parietal_Sup_R | 69.7333±6.1487 | 64.3503±6.2030 | 67.0567±6.2588 | 0.4388 | 0.1003 | 0.7529 | 0.1003 |
| Parietal_Inf_L | 56.2055±6.9412 | 50.8669±7.0714 | 51.4503±5.6839 | 0.7982 | 0.2100 | 0.4079 | 0.4079 |
| Parietal_Inf_R | 58.0314±6.2778 | 52.7797±5.5545 | 53.9759±5.2263 | 0.6444 | 0.1338 | 0.8633 | 0.3515 |
| SupraMarginal_L | 42.7225±4.2273 | 38.0920±4.1873 | 37.7717±5.3869 | 0.5432 | 0.0426 | 0.6209 | 0.9074 |
| SupraMarginal_R | 56.2305±5.9957 | 51.6232±4.5856 | 51.8492±5.8455 | 0.6056 | 0.0441 | 0.8789 | 0.8722 |
| Angular_L | 54.5538±4.7732 | 52.1381±6.0342 | 51.1951±5.8503 | 0.8710 | 0.6123 | 0.7930 | 0.7930 |
| Angular_R | 57.0432±5.9321 | 53.0312±6.3538 | 54.8271±5.5697 | 0.6851 | 0.6249 | 0.9014 | 0.5067 |
| Precuneus_L | 66.4973±5.2228 | 61.4382±5.8365 | 61.2451±6.3001 | 0.7896 | 0.0483 | 0.9682 | 0.9682 |
| Precuneus_R | 77.6199±5.4861 | 73.9463±5.5874 | 72.9021±6.2318 | 0.8250 | 0.3432 | 0.8588 | 0.8588 |
| Paracentral_Lobule_L | 40.0602±7.1469 | 33.0203±7.5225 | 34.3704±5.5322 | 0.4388 | 0.0438 | 0.9401 | 0.4462 |
| Paracentral_Lobule_R | 38.2461±6.4022 | 33.7641±5.2059 | 34.6756±4.5321 | 0.6056 | 0.1440 | 0.6012 | 0.5106 |
| Caudate_L | 28.7668±3.8747 | 29.4290±4.3686 | 27.0122±3.5508 | 0.4388 | 0.3262 | 0.5165 | 0.1359 |
| Caudate_R | 39.0072±3.6011 | 41.8967±4.8071 | 40.6497±3.6820 | 0.4388 | 0.2537 | 0.0246 | 0.2537 |
| Putamen_L | 46.5929±7.4362 | 43.1574±6.7321 | 38.9399±5.4324 | 0.4388 | 0.3492 | 0.3288 | 0.0663 |
| Putamen_R | 43.8527±7.0145 | 43.0648±6.1686 | 43.4433±3.7630 | 0.8704 | 0.9857 | 0.8144 | 0.8144 |
| Pallidum_L | 35.8691±5.9632 | 32.7578±4.6402 | 32.0595±4.8335 | 0.8151 | 0.1770 | 0.8034 | 0.8034 |
| Pallidum_R | 39.7568±5.4487 | 40.3531±5.5373 | 41.2650±3.6958 | 0.6056 | 0.7925 | 0.1245 | 0.4814 |
| Thalamus_L | 26.6981±7.0002 | 24.5839±5.3896 | 20.7260±4.9950 | 0.4388 | 0.8756 | 0.3615 | 0.0417 |
| Thalamus_R | 40.4318±7.1346 | 38.8908±5.2410 | 36.2799±4.4470 | 0.6056 | 0.5672 | 0.5672 | 0.1290 |
| Heschl_L | 37.3570±5.0203 | 35.0436±3.1962 | 34.3327±4.0688 | 0.7937 | 0.3159 | 0.7255 | 0.7255 |
| Heschl_R | 28.2393±4.1493 | 25.5090±4.2872 | 27.6748±4.5709 | 0.4388 | 0.1457 | 0.1359 | 0.1359 |
| Temporal_Sup_L | 55.5952±4.5878 | 50.5372±4.7184 | 50.1879±4.1363 | 0.4388 | 0.0213 | 0.5407 | 0.9582 |
| Temporal_Sup_R | 64.4029±5.4345 | 55.9364±6.5215 | 57.2317±5.8855 | 0.4388 | 0.0051 | 0.2475 | 0.2475 |
| Temporal_Pole_Sup_L | 42.2065±4.3443 | 38.5929±5.0040 | 37.3497±3.3361 | 0.6056 | 0.4313 | 0.4313 | 0.4313 |
| Temporal_Pole_Sup_R | 38.1798±4.3692 | 33.6502±4.3445 | 33.0144±3.7155 | 0.4388 | 0.0348 | 0.3301 | 0.7487 |
| Temporal_Mid_L | 72.5447±4.7073 | 67.9854±5.6186 | 67.5658±5.1058 | 0.6880 | 0.1464 | 0.9925 | 0.9925 |
| Temporal_Mid_R | 66.1459±5.3380 | 58.2261±6.6784 | 59.6089±5.2960 | 0.4388 | 0.0111 | 0.2326 | 0.2326 |
| Temporal_Pole_Mid_L | 33.7829±3.8791 | 31.4165±4.3090 | 31.8798±3.6864 | 0.7937 | 0.5202 | 0.9505 | 0.9505 |
| Temporal_Pole_Mid_R | 32.9373±5.0738 | 30.5247±4.4102 | 30.5455±4.0985 | 0.8426 | 0.7356 | 0.7534 | 0.8549 |
| Temporal_Inf_L | 70.5525±4.3671 | 66.8943±4.9582 | 67.1912±4.5022 | 0.8151 | 0.6508 | 0.6508 | 0.6508 |
| Temporal_Inf_R | 62.0489±4.4248 | 55.6628±6.1965 | 56.6937±4.9733 | 0.4388 | 0.0252 | 0.5814 | 0.3696 |

^a^mean±standard deviation, controlling for age, gender, and education level.

^b^P-values from the permutation-based ANCOVA, controlling for age, gender, and education level between the three groups; the collected P-values were FDR-adjusted over 90 nodes.

^c^P-values from post-hoc tests using a three pairwise permutation-based ANCOVA, controlling for age, gender, and education level; the collected P-values were FDR-adjusted across the three pairwise comparisons.

**Supplementary Table S7.** Correlation between the global network measures and cognitive scores

| Cognitive domain | Score | Network measure | LLD-MCI-A(+) | | LLD-MCI-A(-) | | Group comparison | |
| --- | --- | --- | --- | --- | --- | --- | --- | --- |
|  |  |  | r-value^a^ | P-value^a^ | r-value^a^ | P-value^a^ | Z-value^b^ | P-value^b^ |
| Attention | Digit span test forward | Clustering coefficient | 0.217 | 0.157 | **0.318** | **0.033** | -0.373 | 0.709 |
|  | Trail-making test A z -score | Total strength | 0.296 | 0.064 | **0.571** | **<0.001** | -1.046 | 0.296 |
|  |  | Edge density | 0.081 | 0.620 | **0.377** | **0.018** | -0.959 | 0.338 |
|  |  | Clustering coefficient | 0.077 | 0.638 | **0.388** | **0.015** | -1.010 | 0.312 |
|  |  | Characteristic path length | -0.158 | 0.332 | **-0.467** | **0.003** | 1.055 | 0.292 |
|  |  | Smallworldness | 0.148 | 0.362 | **0.437** | **0.005** | -0.971 | 0.332 |
|  | Trail-making test A (seconds) | Total strength | -0.309 | 0.052 | **-0.519** | **0.001** | 0.777 | 0.437 |
|  |  | Edge density | -0.185 | 0.253 | **-0.391** | **0.014** | 0.687 | 0.492 |
|  |  | Characteristic path length | 0.231 | 0.152 | **0.510** | **0.001** | -0.996 | 0.319 |
|  |  | Smallworldness | -0.091 | 0.577 | **-0.359** | **0.025** | 0.865 | 0.387 |
| Recall and recognition | Word-list recall | Total strength | **0.395** | **0.008** | 0.018 | 0.907 | 1.370 | 0.171 |
|  |  | Characteristic path length | **-0.363** | **0.015** | 0.027 | 0.862 | -1.396 | 0.163 |
|  | Word-list recall z-score | Total strength | **0.375** | **0.012** | 0.004 | 0.979 | 1.337 | 0.181 |
|  |  | Characteristic path length | **-0.319** | **0.035** | 0.061 | 0.692 | -1.342 | 0.180 |
|  | Word-list recognition | Total strength | **0.366** | **0.015** | 0.053 | 0.731 | 1.334 | 0.257 |
|  |  | Characteristic path length | **-0.429** | **0.006** | -0.022 | 0.089 | -1.497 | 0.135 |
|  | Word-list recognition z-score | Total strength | **0.370** | **0.013** | 0.021 | 0.890 | 1.259 | 0.208 |
|  |  | Characteristic path length | **-0.407** | **0.006** | 0.020 | 0.897 | -1.549 | 0.121 |
| Visuospatial function | Constructional praxis | Total strength | **-0.343** | **0.023** | **0.314** | **0.036** | -2.339 | 0.019 |
|  |  | Clustering coefficient | **-0.369** | **0.014** | 0.175 | 0.249 | -1.933 | 0.053 |
|  |  | Characteristic path length | 0.247 | 0.106 | **-0.305** | **0.042** | 1.944 | 0.052 |
|  |  | Smallworldness | **-0.353** | **0.019** | 0.244 | 0.106 | **-2.118** | **0.034** |
|  | Constructional praxis z-score | Total strength | **-0.465** | **0.001** | 0.183 | 0.213 | **-2.360** | **0.018** |
|  |  | Clustering coefficient | **-0.307** | **0.043** | 0.221 | 0.144 | -1.857 | 0.063 |
|  |  | Characteristic path length | **0.413** | **0.005** | -0.097 | 0.525 | 1.839 | 0.066 |
|  |  | Smallworldness | **−0.378** | **0.011** | 0.194 | 0.203 | **-2.036** | **0.042** |

^a^Partial correlation coefficient, controlling for age, gender, and education level.

^b^Z_observed_ and it corresponding P-values for comparing two correlation coefficients.

**Bold** values denote statistical significance at the p<0.05.

**Supplementary Table S8.** Correlation between the nodal network measures and cognitive scores

| Cognitive domain | Score | Network measure | Node | LLD-MCI-A(+) | | LLD-MCI-A(-) | | Group comparison | |
| --- | --- | --- | --- | --- | --- | --- | --- | --- | --- |
|  |  |  |  | r-value^a^ | P-value^a^ | r-value^a^ | P-value^a^ | Z-value^b^ | P-value^b^ |
| Attention | Trail-making test A (seconds) | Clustering coefficient (node) | Right caudate | **0.514** | **0.049** | -0.049 | 0.910 | 1.876 | 0.061 |
|  |  | Regional efficiency | Left middle cingulum | -0.345 | 0.146 | **−0.539** | **0.028** | 0.739 | 0.460 |
| Executive function | COWAT market | Nodal strength | Left inferior orbitofrontal | 0.345 | 0.987 | **0.510** | **0.031** | -0.696 | 0.487 |
|  | COWAT market z-score | Clustering coefficient (node) | Right thalamus | -0.337 | 0.991 | **−0.500** | **0.042** | 0.681 | 0.496 |
|  |  | Regional efficiency | Left inferior orbitofrontal | 0.277 | 0.963 | **0.495** | **0.049** | -0.885 | 0.376 |
|  | COWAT O | Clustering coefficient (node) | Right middle temporal | 0.504 | 0.071 | **0.569** | **0.007** | -0.293 | 0.770 |
| Learning | Word-list learning | Clustering coefficient (node) | Left middle occipital | -0.270 | 0.497 | **−0.533** | **0.015** | 1.088 | 0.277 |
|  | Word-list learning z-score | Nodal strength | Right inferior orbitofrontal | 0.406 | 0.211 | **0.517** | **0.025** | -0.485 | 0.628 |
|  |  | Clustering coefficient (node) | Left middle occipital | -0.288 | 0.478 | **−0.541** | **0.011** | 1.060 | 0.289 |
| Recall and recognition | Word-list recall | Regional efficiency | Right middle cingulum | **0.498** | **0.028** | **0.128** | **0.028** | 1.432 | 0.152 |
|  | Word-list recall z-score | Regional efficiency | Right middle cingulum | **0.477** | **0.046** | **0.063** | **0.046** | 1.563 | 0.118 |
| Visuospatial function | Construction praxis recall | Regional efficiency | Right superior orbitofrontal | 0.177 | 0.251 | **0.495** | **0.049** | -1.247 | 0.213 |

^a^Partial correlation coefficient, controlling for age, gender, and education level; the collected P-values were FDR-adjusted across 90 nodes.

^b^Z_observed_ and it corresponding P-values for comparing two correlation coefficients.

**Bold** values denote statistical significance at the p<0.05.
